# Consensus guidance for monitoring individuals with islet autoantibody-positive pre-stage 3 type 1 diabetes

**DOI:** 10.1007/s00125-024-06205-5

**Published:** 2024-06-24

**Authors:** Moshe Phillip, Peter Achenbach, Ananta Addala, Anastasia Albanese-O’Neill, Tadej Battelino, Kirstine J. Bell, Rachel E. J. Besser, Ezio Bonifacio, Helen M. Colhoun, Jennifer J. Couper, Maria E. Craig, Thomas Danne, Carine de Beaufort, Klemen Dovc, Kimberly A. Driscoll, Sanjoy Dutta, Osagie Ebekozien, Helena Elding Larsson, Daniel J. Feiten, Brigitte I. Frohnert, Robert A. Gabbay, Mary P. Gallagher, Carla J. Greenbaum, Kurt J. Griffin, William Hagopian, Michael J. Haller, Christel Hendrieckx, Emile Hendriks, Richard I. G. Holt, Lucille Hughes, Heba M. Ismail, Laura M. Jacobsen, Suzanne B. Johnson, Leslie E. Kolb, Olga Kordonouri, Karin Lange, Robert W. Lash, Åke Lernmark, Ingrid Libman, Markus Lundgren, David M. Maahs, M. Loredana Marcovecchio, Chantal Mathieu, Kellee M. Miller, Holly K. O’Donnell, Tal Oron, Shivajirao P. Patil, Rodica Pop-Busui, Marian J. Rewers, Stephen S. Rich, Desmond A. Schatz, Rifka Schulman-Rosenbaum, Kimber M. Simmons, Emily K. Sims, Jay S. Skyler, Laura B. Smith, Cate Speake, Andrea K. Steck, Nicholas P. B. Thomas, Ksenia N. Tonyushkina, Riitta Veijola, John M. Wentworth, Diane K. Wherrett, Jamie R. Wood, Anette-Gabriele Ziegler, Linda A. DiMeglio

**Affiliations:** 1 Institute for Endocrinology and Diabetes, National Center for Childhood Diabetes, Schneider Children’s Medical Center of Israel, Petah Tikva, Israel; 2 Faculty of Medical and Health Sciences, Tel Aviv University, Tel Aviv, Israel; 3 Institute of Diabetes Research, Helmholtz Zentrum München, German Research Center for Environmental Health, Munich-Neuherberg, Germany; 4 Forschergruppe Diabetes, Technical University Munich, Klinikum Rechts Der Isar, Munich, Germany; 5 Division of Endocrinology, Department of Pediatrics, Stanford University School of Medicine, Stanford, CA USA; 6 Stanford Diabetes Research Center, Stanford University School of Medicine, Stanford, CA USA; 7 Breakthrough T1D, Gainesville, FL USA; 8 Faculty of Medicine, University of Ljubljana, Ljubljana, Slovenia; 9 Department of Endocrinology, Diabetes and Metabolism, University Medical Centre Ljubljana, Ljubljana, Slovenia; 10 Charles Perkins Centre and Faculty of Medicine and Health, University of Sydney, Sydney, NSW Australia; 11 JDRF/Wellcome Diabetes and Inflammation Laboratory, Wellcome Centre Human Genetics, Nuffield Department of Medicine Oxford NIHR Biomedical Research Centre, University of Oxford, Oxford, UK; 12 Department of Paediatrics, University of Oxford, Oxford, UK; 13 Center for Regenerative Therapies Dresden, Faculty of Medicine, Technical University of Dresden, Dresden, Germany; 14 Paul Langerhans Institute Dresden, Helmholtz Centre Munich at the University Clinic Carl Gustav Carus of TU Dresden and Faculty of Medicine, Dresden, Germany; 15 The Institute of Genetics and Cancer, University of Edinburgh, Edinburgh, UK; 16 Department of Public Health, NHS Fife, Kirkcaldy, UK; 17 Robinson Research Institute, The University of Adelaide, Adelaide, SA Australia; 18 Adelaide Medical School, The University of Adelaide, Adelaide, SA Australia; 19 Division of Paediatrics, Women’s and Children’s Hospital, Adelaide, SA Australia; 20 Discipline of Paediatrics & Child Health, School of Clinical Medicine, UNSW Medicine & Health, Sydney, NSW Australia; 21 Breakthrough T1D, Lisbon, Portugal; 22 International Society for Pediatric and Adolescent Diabetes (ISPAD), Berlin, Germany; 23 Diabetes & Endocrine Care Clinique Pédiatrique (DECCP), Clinique Pédiatrique/Centre Hospitalier (CH) de Luxembourg, Luxembourg City, Luxembourg; 24 Faculty of Science, Technology and Medicine, University of Luxembourg, Esch-Belval, Luxembourg; 25 Department of Pediatrics, Barbara Davis Center for Diabetes, University of Colorado Anschutz Medical Campus, Aurora, CO USA; 26 Department of Clinical and Health Psychology, University of Florida, Gainesville, FL USA; 27 Department of Pediatrics, University of Florida Diabetes Institute, Gainesville, FL USA; 28 Breakthrough T1D, New York, NY USA; 29 T1D Exchange, Boston, MA USA; 30 Department of Clinical Sciences, Malmö, Lund University, Lund, Sweden; 31 Department of Pediatrics, Skåne University Hospital, Malmö and Lund, Sweden; 32 Children’s Diabetes Foundation, Aurora, CO USA; 33 American Diabetes Association, Arlington, VA USA; 34 NYU Langone Medical Center, New York, NY USA; 35 Center for Interventional Immunology and Diabetes Program, Benaroya Research Institute, Seattle, WA USA; 36 Sanford Research, Sioux Falls, SD USA; 37 Department of Pediatrics, Sanford School of Medicine, University of South Dakota, Sioux Falls, SD USA; 38 Pacific Northwest Diabetes Research Institute, University of Washington, Seattle, WA USA; 39 Division of Endocrinology, University of Florida College of Medicine, Gainesville, FL USA; 40 School of Psychology, Deakin University, Geelong, VIC Australia; 41 The Australian Centre for Behavioural Research in Diabetes, Diabetes Victoria, Carlton, VIC Australia; 42 Institute for Health Transformation, Deakin University, Geelong, VIC Australia; 43 Department of Paediatrics, University of Cambridge and Cambridge University Hospitals NHS Foundation Trust, Cambridge Biomedical Campus, Cambridge, UK; 44 Human Development and Health, Faculty of Medicine, University of Southampton, Southampton, UK; 45 National Institute for Health and Care Research Biomedical Research Centre, University Hospital Southampton NHS Foundation Trust, Southampton, UK; 46 Mount Sinai South Nassau, Oceanside, NY USA; 47 Department of Pediatrics, Indiana University School of Medicine, Indianapolis, IN USA; 48 Department of Behavioral Sciences and Social Medicine, Florida State University College of Medicine, Tallahassee, FL USA; 49 Association of Diabetes Care & Education Specialists, Chicago, IL USA; 50 Medical Psychology, Hannover Medical School, Hannover, Germany; 51 Endocrine Society, Washington, DC USA; 52 Division of Pediatric Endocrinology and Diabetes, University of Pittsburgh, UPMC Children’s Hospital of Pittsburgh, Pittsburgh, PA USA; 53 Department of Pediatrics, Kristianstad Hospital, Kristianstad, Sweden; 54 Department of Pediatrics, Cambridge University Hospitals NHS Foundation Trust, Cambridge, UK; 55 Department of Endocrinology, UZ Gasthuisberg, KU Leuven, Leuven, Belgium; 56 Department of Family Medicine, Brody School of Medicine, East Carolina University, Greenville, NC USA; 57 Department of Internal Medicine, Division of Metabolism, Endocrinology and Diabetes, University of Michigan, Ann Arbor, MI USA; 58 Center for Public Health Genomics, University of Virginia, Charlottesville, VA USA; 59 Department of Pediatrics, University of Florida, Gainesville, FL USA; 60 Division of Endocrinology, Long Island Jewish Medical Center, Northwell Health, Donald and Barbara Zucker School of Medicine at Hofstra/Northwell, New Hyde Park, NY USA; 61 Division of Pediatric Endocrinology and Diabetology, Herman B Wells Center for Pediatric Research, Center for Diabetes and Metabolic Diseases, Indiana University School of Medicine, Indianapolis, IN USA; 62 Diabetes Research Institute, University of Miami Miller School of Medicine, Miami, FL USA; 63 Cincinnati Children’s Hospital Medical Center, Cincinnati, OH USA; 64 NIHR Clinical Research Network Thames Valley and South Midlands, Oxford, UK; 65 Division of Endocrinology and Diabetes, Baystate Children’s Hospital and University of Massachusetts Chan Medical School - Baystate, Springfield, MA USA; 66 Research Unit of Clinical Medicine, Department of Pediatrics, Medical Research Center, Oulu University Hospital and University of Oulu, Oulu, Finland; 67 The Walter and Eliza Hall Institute of Medical Research, Parkville, VIC Australia; 68 Department of Diabetes and Endocrinology, Royal Melbourne Hospital, Parkville, VIC Australia; 69 Department of Paediatrics, The Hospital for Sick Children, University of Toronto, Toronto, ON Canada; 70 Department of Pediatric Endocrinology, Rainbow Babies and Children’s Hospital, University Hospitals Cleveland Medical Center, Cleveland, OH USA

**Keywords:** Autoantibodies, Glucose monitoring, Prevention, Type 1 diabetes

## Abstract

Given the proven benefits of screening to reduce diabetic ketoacidosis (DKA) likelihood at the time of stage 3 type 1 diabetes diagnosis, and emerging availability of therapy to delay disease progression, type 1 diabetes screening programmes are being increasingly emphasised. Once broadly implemented, screening initiatives will identify significant numbers of islet autoantibody-positive (IAb^+^) children and adults who are at risk of (confirmed single IAb^+^) or living with (multiple IAb^+^) early-stage (stage 1 and stage 2) type 1 diabetes. These individuals will need monitoring for disease progression; much of this care will happen in non-specialised settings. To inform this monitoring, JDRF in conjunction with international experts and societies developed consensus guidance. Broad advice from this guidance includes the following: (1) partnerships should be fostered between endocrinologists and primary-care providers to care for people who are IAb^+^; (2) when people who are IAb^+^ are initially identified there is a need for confirmation using a second sample; (3) single IAb^+^ individuals are at lower risk of progression than multiple IAb^+^ individuals; (4) individuals with early-stage type 1 diabetes should have periodic medical monitoring, including regular assessments of glucose levels, regular education about symptoms of diabetes and DKA, and psychosocial support; (5) interested people with stage 2 type 1 diabetes should be offered trial participation or approved therapies; and (6) all health professionals involved in monitoring and care of individuals with type 1 diabetes have a responsibility to provide education. The guidance also emphasises significant unmet needs for further research on early-stage type 1 diabetes to increase the rigour of future recommendations and inform clinical care.

**Graphical Abstract:**

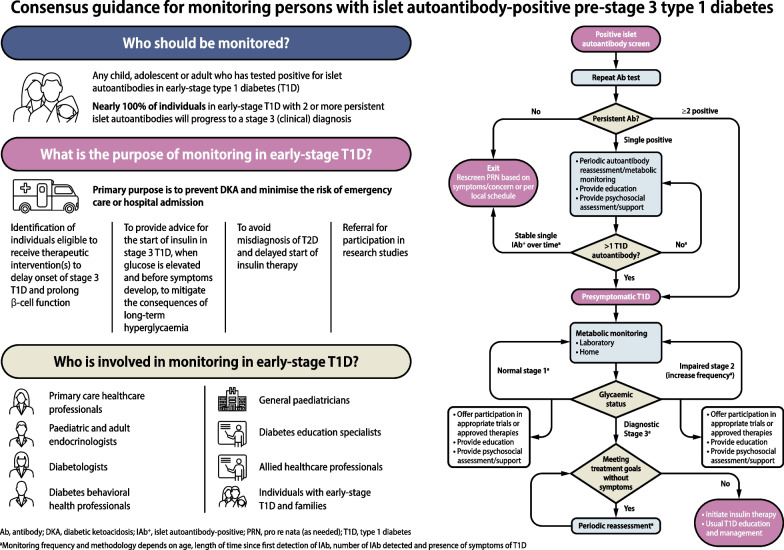

**Supplementary Information:**

The online version contains peer-reviewed but unedited supplementary material including a slideset of the figures for download, available at 10.1007/s00125-024-06205-5.

## Overview

Currently, screening of individuals for islet autoantibodies is undertaken as part of programmes to detect children, adolescents and adults who are at higher risk of developing type 1 diabetes due to having a first-degree relative with type 1 diabetes or having a known high-risk HLA genotype. Periodic monitoring of people who have screened positive for one or more autoantibodies (islet autoantibody-positive [IAb^+^] individuals) is largely, but not always, conducted within these cohort studies. However, up to 90% of people who develop type 1 diabetes are not part of at-risk groups. Thus, screening programmes within the general population are being initiated and guidance for monitoring in non-specialist settings is urgently needed. The guidance provided here was developed by a series of expert working groups, convened as part of a JDRF initiative to document the aims, scope and purpose of monitoring for children, adolescents and adults with islet autoantibody positivity, along with recommended frequencies of monitoring and actions for healthcare professionals (HCPs) when risk of progression towards symptomatic type 1 diabetes is high. This includes expert clinical advice for educational and psychosocial support for IAb^+^ individuals, including for their families and caregivers. The expert clinical advice for adults reflects available data, yet it is important to note that there are very limited data in adults aged 45 years and older who are IAb^+^. It is also important to note that this consensus document does not encompass screening for islet autoantibodies, and only provides expert clinical advice for monitoring of individuals who have screened positive for at least one islet autoantibody.

## Introduction and rationale

The presence of islet autoantibodies for a presymptomatic period of variable duration in first-degree relatives of individuals with type 1 diabetes has been known for more than 40 years [1], with recommendations for islet autoantibody screening appearing soon after [2]. Decades of subsequent research and monitoring of individuals with islet autoantibody positivity has led to the paradigm shift that type 1 diabetes is a continuum of stages, from genetic risk through to autoimmunity and then metabolic disease. This has been accompanied by the evolution of descriptive terminology that reflects these stages (Table 1). Similarly, treatment options have moved on from monitoring and managing metabolic disease to include options for modulating the autoimmune response [3, 4].

**Table 1:** Staging criteria for autoantibody-positive individuals in pre-stage 1 and stage 1–3 type 1 diabetes [16–18]

Stage of T1D	Islet autoantibody status	Glycaemic status	Symptoms	Insulin required
At-risk (pre-stage 1 T1D)	Single autoantibody or transient single autoantibody	• Normoglycaemia • FPG <5.6 mmol/l (<100 mg/dl) • 120 min OGTT <7.8 mmol/l (<140 mg/dl) • HbA_1c_ <39 mmol/mol (<5.7%)	No symptoms	Not required
Stage 1 T1D (also referred to as early-stage T1D or presymptomatic T1D)	≥2 autoantibodies	• Normoglycaemia • FPG <5.6 mmol/l (<100 mg/dl) • 120 min OGTT <7.8 mmol/l (<140 mg/dl) • HbA_1c_ <39 mmol/mol (<5.7%)	No symptoms	Not required
Stage 2 T1D (also referred to as early-stage T1D or presymptomatic T1D)	≥2 autoantibodies^a^	Glucose intolerance or dysglycaemia not meeting diagnostic criteria for stage 3 T1D, with at least two of the following, or meeting the same single criteria at two time points within 12 months: • FPG 5.6–6.9 mmol/l (100–125 mg/dl) • 120 min OGTT 7.8–11.0 mmol/l (140–199 mg/dl) • OGTT values ≥11.1 mmol/l (≥200 mg/dl) at 30, 60 and 90 min • HbA_1c_ 39–47 mmol/mol (5.7–6.4%) or longitudinal ≥10% increase in HbA_1c_ [66, 67] from the first measurement with stage 2 T1D • CGM values >7.8 mmol/l (>140 mg/dl) for 10% of time over 10 days’ continuous wear [73]^b^, and confirmed by at least one other non-CGM glucose measurement test listed	No symptoms	Not required
Stage 3 T1D	≥1 autoantibody	Persistent hyperglycaemia with or without symptoms, as measured and confirmed by one or more of the following: • one random venous glucose ≥11.1 mmol/l (≥200 mg/dl) with overt symptoms • 120 min OGTT ≥11.1 mmol/l (≥200 mg/dl) and/or • two random venous glucose ≥11.1 mmol/l (≥200 mg/dl) and/or • FPG ≥7.0 mmol/l (≥126 mg/dl) and/or • laboratory-tested HbA_1c_ ≥48 mmol/mol (≥6.5%) • CGM values >7.8 mmol/l (>140 mg/dl) for 20% of time over 10 days’ continuous wear [73]^b^ and confirmed by at least one other non-CGM glucose measurement test listed	May include^c^: • Polyuria • Polydipsia • Weight loss • Fatigue • DKA	+/- Insulin, based on glycaemic status

^a^Some people with confirmed persistent prior multiple autoantibody positivity may revert to single autoantibody status or negative status [95]

^b^CGM is ideally blinded and must be applied and interpreted by a trained HCP. Note, use of CGM-derived criterion did not achieve consensus within the consensus panel and CGM metrics are not part of current ADA or ISPAD guidelines on staging criteria in type 1 diabetes [16, 155]

^c^Stage 3 might not include symptoms

FPG, fasting plasma glucose; T1D, type 1 diabetes

Screening programmes have developed to the point that large numbers of children and adults at risk of and with early-stage type 1 diabetes have been intensively followed in longitudinal cohort studies [5–15] centred on understanding the natural history of progression to symptomatic type 1 diabetes (see Table 2 for a list of studies available for participation). Of note, many entry criteria for individuals with presymptomatic type 1 diabetes into these studies require a family history of type 1 diabetes or HLA genetic risk, and most are focused on paediatric populations. Based on the outcomes of these and other studies, stages of presymptomatic and symptomatic type 1 diabetes are now clinically defined (Table 1) to a degree of clinical consensus [16–18], although regulatory agencies and research studies may differ in definitions. Using these classifications, individuals can be monitored, diagnosed with diabetes and even, at times, started on insulin replacement therapy early in the disease course, based on meeting American Diabetes Association (ADA) [18], International Society for Pediatric and Adolescent Diabetes (ISPAD) [16] or American Association of Clinical Endocrinology (AACE) [19] diagnostic criteria. To date, the ISPAD guidelines have provided metabolic and autoantibody monitoring recommendations for children with presymptomatic type 1 diabetes [16], but do not make specific recommendations for education or psychosocial support in IAb^+^ individuals, monitoring of single IAb^+^ individuals or when to start insulin. The Fr1da study has suggested and introduced specific recommendations for children [20]. A separate set of recommendations based on a Delphi-survey of expert opinion has provided guidance on metabolic and autoantibody monitoring, with recommendations for education and psychosocial support, but does not specifically address adults with early-stage type 1 diabetes [21]. Consequently, to date there is no available guidance on monitoring in adults or in individuals with single islet autoantibody positivity, or on when insulin therapy is indicated.

**Table 2:** Established population-based screening and monitoring studies in early-stage type 1 diabetes

Acronym	Study name/description
ASK	Autoimmunity Screening for Kids programme [7]
BABYDIAB	Part of the international Type 1 Data Intelligence (T1DI) project [156]
DAISY	Diabetes Autoimmunity Study in the Young [6]
DIPP	Type 1 Diabetes Prediction and Prevention Study based in Finland [11]
DPT-1	Diabetes Prevention Trial–Type 1 [12]
ENDIT	European Nicotinamide Diabetes Intervention Trial [13]
Fr1da	Population-based healthcare research study based in Bavaria, Germany [9]
INNODIA	Global partnership between academic institutions, commercial partners and patient organisations [14]
PLEDGE	Population Level Estimation of T1D Risk Genes in Children [155]
TEDDY	The Environmental Determinants of Diabetes in the Young study [5]
Type 1 Diabetes TrialNet	International research network centred on delaying or preventing T1D [10]
Type1Screen	Australian screening and monitoring programme open to relatives of individuals with type 1 diabetes and IAb^+^ people identified through other screening pathways (ANZCTR registration no. ACTRN12620000510943)

Note that major research networks are included in the table, but this is not an exhaustive list

ANZCTR, Australian New Zealand Clinical Trials Registry

Consensus on evidence-based expert clinical advice for monitoring is an important unmet need since a positive test for islet autoantibodies (Table 3) is a condition for access to disease-modifying therapies, such as teplizumab [22]. In addition, islet autoantibody screening is anticipated to become more common [7, 23–25], highlighting the need for clear monitoring advice.

**Table 3:** Autoantibodies against islet autoantigens detected in stage 1–3 type 1 diabetes

Autoantibody	Islet specificity	Typical characteristics
IAA	Insulin	• Common as a first detected autoantibody in young children [157, 158] • Appearance is more common in younger children [159] • Frequency of appearance declines with age • Not informative for individuals treated with insulin, who often develop antibodies in response to injected insulin
GADA	GAD	• Common as a first detected autoantibody in childhood, up until age 15 years [157, 158, 160] • Adult-onset cases most often present with GADA [161] • Is associated with slower progression to T1D [162] and is often found as a single positive islet autoantibody, especially in adults
IA-2A (also known as ICA512)	Tyrosine phosphatase islet antigen-2	Presence is associated with more-advanced islet autoimmunity and faster progression to stage 3 T1D [55, 163]
ZnT8A	Zinc transporter type 8, a transmembrane protein in the beta cell granule	Presence can improve risk stratification in individuals with single GADA^+^, IAA^+^ or IA-2A^+^ status [164]
ICA	Multiple antigens, undefined	Detected by indirect immunofluorescence on islet cell tissue. While not frequently measured other than in research studies, it does add to risk determination in the presence of other biochemical autoantibodies

IA-2A, insulinoma antigen-2 autoantibody; ICA512, islet cell autoantigen 512; T1D, type 1 diabetes

Screening efforts are identifying an ever-growing number of IAb^+^ people who warrant education and ongoing monitoring for progression towards clinical diabetes. Evidence shows that such monitoring in research studies can significantly reduce the incidence of diabetic ketoacidosis (DKA) at diagnosis [24, 26–33], occurring in up to 70% of unmonitored individuals, which is greatly lowered for individuals participating in follow-up studies [26, 34–39]. The impact of monitoring in general clinical practice on DKA rates is not known. DKA is a life-threatening condition that requires hospital admission, with significant associated costs for critical care [40–42]. Additionally, in a number of studies, DKA at presentation of type 1 diabetes in youths has been associated with higher HbA_1c_ that was sustained for up to 11 years after diagnosis [43–45]. Other studies have, however, not found such an association between DKA at presentation of type 1 diabetes and higher long-term glycaemic levels [46]. The lack of DKA at onset of type 1 diabetes is also predictive of fewer severe hypoglycaemic events 10 years after diagnosis [47]. In this context, the overall goals of monitoring are described in Table 4.

**Table 4:** Purpose of monitoring in IAb^+^ children, adolescents and adults

1. Primary purpose is to prevent DKA and to minimise the risk of requiring emergency care or hospital admission
2. Identification for and monitoring of therapeutic intervention(s) to delay stage 3 T1D onset (where available) and prolong beta cell function
3. To provide advice for the start of insulin in stage 3 T1D, when glucose is sufficiently elevated and before symptoms develop, to optimise HbA_1c_ and avoid the consequences of hyperglycaemia on long-term glycaemic outcomes
4. To avoid misdiagnosis of T2D and delayed commencement of insulin therapy
5. Referral for participation in research studies

T1D, type 1 diabetes; T2D, type 2 diabetes

Monitoring of people with islet autoantibody positivity outside of research settings will require expert clinical advice that is clear and actionable by HCPs who have limited expertise in diabetes. As indicated, current insights into monitoring progression to clinical type 1 diabetes are largely derived from research studies of individuals known to be at risk of type 1 diabetes, and general population data are less extensive. With this caveat, knowledge on best practices is particularly important for primary-care and secondary-care physicians who may not frequently see people known to be at risk of type 1 diabetes, and yet who will be tasked with the initial aspects of monitoring following a positive autoantibody screen. Other people who may assist with care of these individuals will include nurse practitioners, physician assistants, diabetes care and education specialists (DCES), psychologists and other mental and behavioural health professionals, all of whom have a role in supporting IAb^+^ individuals and their families within the monitoring environment. Clear expert clinical advice for monitoring by these groups of HCPs increases the likelihood that individuals at risk for or in early stages of type 1 diabetes, and their families, can receive accurate and actionable education about presymptomatic type 1 diabetes and their individual status.

### The requirement for monitoring

Islet autoantibodies against four major pancreatic autoantigens are currently clinically available; these consist of IAA, GADA, insulinoma antigen-2 autoantibody (IA-2A; also called islet cell autoantigen 512 [ICA512]) and ZnT8A [48]. These are often considered ‘biochemical autoantibodies’ and are the screening targets recommended by the most-recent ADA Standards of Care [25]. A further islet autoantibody assay, for ICA, using indirect immunofluorescence on pancreatic tissue, has been used for screening purposes, but it is less available outside of research studies and the antigenic targets are not fully known. Considerable evidence in multiple populations supports the concept that the number and type of biochemical autoantibodies can be used to predict risk for progression to clinical disease (stage 3 type 1 diabetes; see Table 1). These autoantibodies and their characteristics are described in Table 3. However, it must be noted that these attributes are derived from observations made in known IAb^+^ populations in the research environment. Further data from studies in IAb^+^ groups in the general population are needed.

Confirmation of IAb^+^ status is important to identify the persistence of the underlying autoimmune response and the validity of the target antigen, although the accuracy of autoantibody tests can vary between laboratories and between target antigens. Therefore, the first positive test should be confirmed with a second test within 3 months [49] and, where possible, in a laboratory that meets the performance standards set by the Islet Autoantibody Standardization Program (IASP) [50]. Persistent IAb^+^ status on two or more different samples is needed, using sensitive and specific assays with high predictive value for disease progression [51]. Several research programmes have tested for islet autoantibody status using capillary sampling to obtain serum or dried blood spots for assessment; however, venous samples are preferred (due to reduced interference from haemolysis) and should be used as confirmation whenever capillary testing has been performed initially.

Predicting when an individual with type 1 diabetes-related autoantibodies may progress to stage 3 type 1 diabetes is difficult. However, in children and adolescents, persistent multiple IAb^+^ status confirms early-stage (stage 1 or stage 2) type 1 diabetes with higher rate of progression to stage 3 type 1 diabetes compared with single IAb^+^ status [52]. For the same reasons as discussed for single IAb^+^ status, confirmation of multiple IAb^+^ status is important, as it indicates early-stage type 1 diabetes, and should adhere to the ‘rule of twos’, i.e. the presence of two different autoantibodies, confirmed in two tests from two separate samples [51–54]. Subsequent loss of individual antibodies is not associated with a slower rate of progression. The type of positive autoantibody (Table 3) is also of importance, since, as children age, relative risks for progression with each antibody type will change [55, 56], with some evidence that this is also true for adults [55, 57]. Consideration of these data, along with autoantibody titres, may aid risk stratification [58]. Although fewer data are available in adults, Type 1 Diabetes TrialNet cohort data indicate that the rate of progression to type 1 diabetes in IAb^+^ adults is slower than in children [59].

Misdiagnosis of type 1 diabetes as type 2 diabetes in adolescents and adults can lead to DKA [60], as this misdiagnosis means that these individuals are often not started on insulin [61]. Latent autoimmune diabetes of adults (LADA) can also be misdiagnosed as type 2 diabetes [62], with a risk of delayed insulin initiation. These observations emphasise the value of autoantibody testing for newly-diagnosed adults with diabetes, particularly when they have features of type 1 diabetes (e.g. younger age, non-obese, sudden weight loss, mild acidosis, DKA, hyperglycaemia >16.7 mmol/l [>300 mg/dl]) [63], for making an accurate diagnosis and starting appropriate treatment. It is, however, important to recognise that some individuals with new-onset type 1 diabetes have a phenotype that does not differ substantially from people with type 2 diabetes, particularly given the increased prevalence of obesity [60, 64]. Misdiagnosis of MODY is also reported [65], suggesting that islet autoantibody screening can be valuable at presentation of all forms of diabetes.

An important outcome of monitoring individuals with islet autoantibody positivity is to inform the decision to initiate insulin therapy, and this is an area of evolving practice. In some centres, individuals with hyperglycaemia (see Table 5) but with HbA_1c_ <48 mmol/mol (<6.5%) might not be started on insulin without the presence of symptoms. Sequential HbA_1c_ monitoring has been productive in this context in paediatric studies on individuals with islet autoantibody positivity, since an absolute ≥10% increase from baseline, even if the HbA_1c_ test reading stays below 48 mmol/mol (6.5%), is predictive of disease progression [66, 67] within a median of 1 year. Risk of progression within 2 years following a confirmed ≥10% increase in HbA_1c_ is lower for older individuals. This aspect of stage 3 type 1 diabetes (i.e. when to start insulin once hyperglycaemia is confirmed) requires further evidence to support clinical practice, to better understand the metabolic and mental-health outcomes.

**Table 5:** Attributes of current monitoring methods

Method	Pros	Cons	Metrics obtained
Reference OGTT^a^	• Gold standard in research settings • Used to stage disease and predict progression	• Requires glucose load and 2–5× blood draws over 2 h	• Glycaemic staging • Risk scores for progression (DPTRS, DPTRS60, Index60, M60, M120, PLS) [94, 165–169]
Standard OGTT^b^	• Similar to test for GDM: OGTT with 2× blood draws (compared with 3× draws in GDM test), performed routinely in clinical care	• Requires 2× blood draws: fasting and at 2 h	• 120 min OGTT-derived glucose • M120
Random glucose	• One-off sample • Low cost	• Requires a blood draw or fingerstick test • Less sensitive than 120 min OGTT	• Similar to 120 min OGTT-derived glucose [96] if obtained 2 h postprandially
Standard HbA_1c_ test	• Highly specific for clinical diagnosis of stage 3 T1D • Can use capillary sample • Longitudinal HbA_1c_ may be as informative as OGTT [66]	• Indicates 3 month mean glucose. Often normal in asymptomatic or recent-onset stage 3 T1D • May be affected by age, non-diabetes disease states (e.g. renal, haematological syndromes) • Not suitable in the home setting	• Risk of progression to ‘clinical disease’: HbA_1c_ >39 mmol/mol (>5.7%) [170] • 10% rise from baseline (at first positive islet autoantibody) over 3–12 months [66, 67], suggests dysglycaemia and progression to stage 2 T1D • Consider use of CGM if 10% rise in HbA_1c_ is confirmed, or higher frequency of SMBG, to monitor risk for progression
CGM^c^	• Can be used at home • Can be blinded for physician review only in some regions • Optimal duration of CGM wear is validated in adults and children >2 years of age with diagnosed T1D, at all glycaemic levels [171]	• Risk of anxiety for unblinded user seeing CGM fluctuations and experiencing alarms • Requires appropriate education on use and interpretation • Many primary-care HCPs are unfamiliar with interpretation • Cost and access issues • Duration of wear not validated in early-stage T1D	• Sensitive in detecting individuals with asymptomatic stage 3 T1D and dysglycaemia in stage 2 T1D [73] • Risk of progression to ‘clinical disease’, i.e. 10% of time with glucose >7.8 mmol/l (>140 mg/dl) has been associated with an 80% risk of progression to T1D within 12 months [72] • ≥5% time with glucose ≥7.8 mmol/l (≥140 mg/dl) has been associated with a 40% risk of progression to T1D within 2 years [71] • Other PPV metrics not tested
SMBG	• Simple to use at home • Comparatively low cost	• Uncomfortable for users, can affect accuracy and use • Optimal timing and frequency have not been determined	• Immediate capillary blood glucose test result • 2 h postprandial measure likely of most value
C-peptide	• Validated measure of beta cell function • Stimulated C-peptide in research settings is valuable to assess insulin production and distinguish between T1D (or stages of T1D) and T2D	• Can be falsely low in hypoglycaemia <3.9 mmol/l (<70 mg/dl), in severe hyperglycaemia/DKA or after fasting, so concomitant serum glucose should be checked for interpretation • Wide range of values at clinical diagnosis, including >0.2 nmol/l, and persistent, but low, levels of secretion can be seen long after diagnosis • Presence of C-peptide does not exclude T1D and on its own is not useful for staging or diagnosis of T1D	• A stimulated postprandial C-peptide value ≤0.2 nmol/l with IAb^+^ status can assist with appropriately classifying diabetes type
Repeat antibody testing	• Confirms initial IAb^+^ test result and progression to multiple IAb^+^ status	• None	• Autoantibody type and single IAb^+^ or multiple IAb^+^ status
Education	• Provides awareness of diabetes symptoms and signs	• None	• Person-reported outcomes for possible progression to stage 3 T1D

^a^Used in research settings for staging progression of impaired glucose tolerance as C-peptide provides important predictive value

^b^Used in clinical practice to detect impaired glucose tolerance in prediabetes and gestational diabetes mellitus

^c^Use of CGM-derived criterion did not achieve consensus within the consensus panel, with further evidence required to confirm findings to date

DPTRS, Diabetes Prevention Trial-Type 1 risk score; GDM, gestational diabetes mellitus; M60, 60 min test result; M120, 120 min test result; PLS, partial least squares; PPV, positive predictive value; T1D, type 1 diabetes; T2D, type 2 diabetes

#### What should be monitored?

It is acknowledged that the practice of monitoring of individuals with islet autoantibody positivity must accommodate different settings with diverse healthcare resources. In this context, there are multiple available tools for monitoring, including self-monitored blood glucose (SMBG), periodic continuous glucose monitoring (CGM), a standard OGTT, random venous glucose, HbA_1c_ and repeat islet autoantibody monitoring. In this context, serial stimulated C-peptide measurement during an OGTT can be used to assess deterioration of beta cell function and to predict risk development of type 1 diabetes [68]. Since individuals who present with clinical type 1 diabetes (stage 3) often have significant residual beta cell function [69], they may benefit from therapies that can optimise prolongation of insulin secretion [70].

The pros and cons of each monitoring method are documented in Table 5. Identification of an increase in sequential HbA_1c_ values from a baseline reading can be as informative as 2 h OGTT values in predicting risk of stage 3 type 1 diabetes in youth with genetic risk and type 1 diabetes-associated autoantibodies [66, 67]. Ongoing research continues to evaluate the role of CGM (including professional CGM, which is blinded to the user) in aiding in the identification of individuals, including those with a normal OGTT, who are likely to rapidly progress to stage 3 type 1 diabetes [71–73]. To date, use of CGM metrics in individuals who have multiple IAb^+^ status has been shown to be predictive of progression to type 1 diabetes, but CGM measures are not yet as sensitive as OGTT testing [74].

#### Where should monitoring take place?

In practice, monitoring should be carried out wherever the skills and resources exist to perform the appropriate tests (Table 5). However, since many people will be monitored in primary care, there is a need to consider different intensities of monitoring consistent with resources available. The capabilities of primary-care HCPs and other care providers should be applied to monitoring of early-stage type 1 diabetes without the need to refer to an expert practitioner, until clinically appropriate. In primary care, this may help specify basic education about symptoms and glycaemic signposts. It is understood that, compared with stage 1, monitoring in stage 2 type 1 diabetes may require more-expert practitioners.

## Objectives and methodology

The aim of this international consensus report is to formulate expert clinical advice, based on current evidence and expert opinion, that specifies the required monitoring and management approach for people who have been identified as having IAb^+^ status and pre-stage 3 type 1 diabetes, and can be used in daily clinical practice. Overall, these key principles should encompass: (1) who should be monitored; (2) which endpoints to monitor; (3) the frequency and duration of monitoring; (4) initiation of insulin during stage 3 type 1 diabetes; and (5) how to provide psychosocial and educational support for affected individuals and families.

We acknowledge that monitoring of IAb^+^ individuals will occur in diverse settings, with variable resources to support effective monitoring of IAb^+^ individuals. Thus, a guiding principle of this consensus report is to provide advice that is straightforward and actionable within the landscape of available clinical skills and resources, wherever the monitoring will take place. The audience for this consensus document, therefore, includes: (1) primary-care providers; (2) endocrinologists and diabetologists; (3) DCES; (4) mental and behavioural health professionals; and (5) individuals at risk for or in early stages of type 1 diabetes and their families.

### Methodology

The consensus process was initiated by the JDRF with a conference held on 21 February 2023 at the 16th International Conference on Advanced Technologies & Treatments for Diabetes (ATTD) in Berlin, Germany, with in-person or virtual attendance. MP served as Chair of the project and LAD served as Vice Chair. A mission statement was created and the attendees were invited by email from JDRF and the consensus project leadership. The initial working group comprised 61 internationally recognised physicians, nurse practitioners, clinical psychologists and DCES, with expertise in the diagnosis and care of people with early-stage type 1 diabetes. The conference was centred on monitoring of IAb^+^ people in early-stage type 1 diabetes, including discussions of current guidance on current best practice for monitoring, as applied by several prospective type 1 diabetes prevention trials (discussed in detail below).

Following a moderated discussion, expert participants were offered the opportunity to join at least one of four working groups, each focused on key aspects of monitoring. Each working group was chaired by two expert contributors, as noted below, and was tasked with self-organised review of the available evidence, participation in serial online discussions and development of core principles. The working groups were: (1) monitoring in children and adolescents (Chairs: REJB and KJG); (2) monitoring in adults (Chairs: RS-R and JMW); (3) educational needs (Chairs: KJB and BIF); and (4) psychosocial interventions (Chairs: KAD and LBS). This subsequently generated 21 separate online group discussions. Each aspect of these discussions was documented with support from JDRF team members and a medical writer. It must be noted that this document is not intended or structured as a systematic review.

**Fig. 1:**
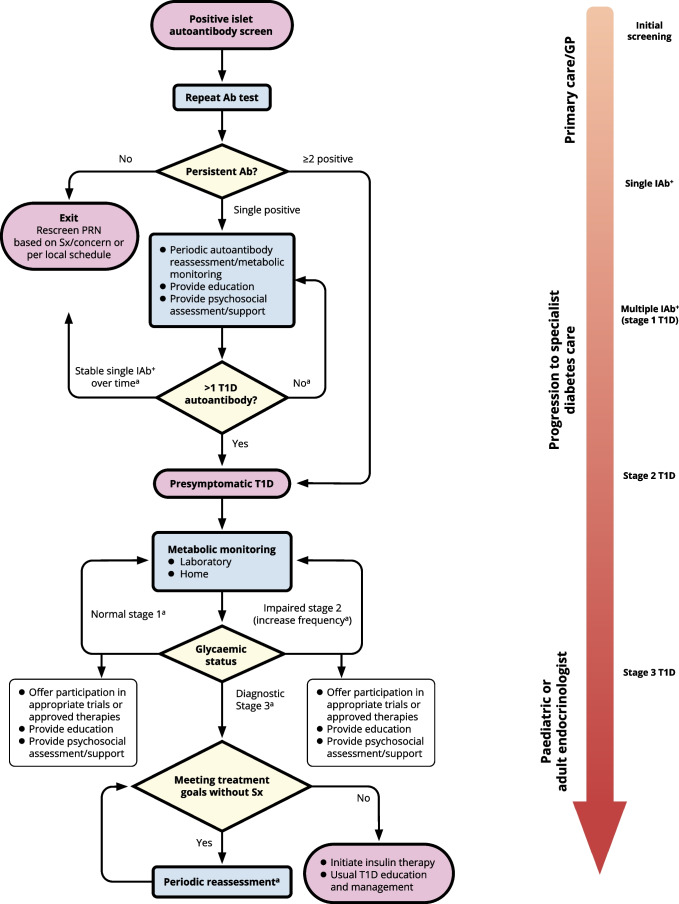
Algorithm for monitoring of people screened positive for one or more islet autoantibodies. ^a^Monitoring frequency and methodology depends on age, length of time since first detection of islet autoantibody, number of islet autoantibodies detected and presence of symptoms of type 1 diabetes (see Tables 1, 3, 4 and 5). Ab, antibody; GP, general practitioner; PRN, pro re nata (as needed); Sx, symptoms; T1D, type 1 diabetes. This figure is available as part of a downloadable slideset

On a weekly basis, from 3 May 2023 onwards, evidence-based statements and expert interpretations were drafted for review and revision. At the end of this iterative process, an agreed narrative review of the available evidence was compiled along with the expert clinical advice. Each bulleted principle was assigned a level of supporting evidence (A, B, C or E; see electronic supplementary material [ESM] Table 1) that adheres to the evidence-grading system for ‘*Standards of Care in Diabetes—2023*’, published by the ADA [75]. The process concluded with a conference to review and endorse the penultimate consensus report at the ADA’s 83rd Scientific Sessions in San Diego, CA, USA. Following this meeting, a revised draft was made available for public comment, after which the consensus document was finalised. The outcomes of this process are also summarised in an algorithm that details the decision path for monitoring of IAb^+^ people regardless of whether they were screened as part of a research protocol or in the clinical setting for any reason (Fig. 1).

## 1. Terminology

Precise and consistent language is important to facilitate clear communication and education. As the field has evolved, so has the language around multiple IAb^+^ status, the stages of type 1 diabetes and associated risk of progression. It was once commonplace to refer to ‘risk of’ and ‘prevention of’ type 1 diabetes in individuals with multiple IAb^+^ status. However, the staging criteria recognise seroconversion to multiple IAb^+^ status as the onset of early-stage type 1 diabetes and, thus, it is not possible to both have a condition and be ‘at risk’ for it.

Therefore, stage 1 type 1 diabetes and stage 2 type 1 diabetes (Table 1) should be referred to by their defined names or collectively referred to as ‘early-stage type 1 diabetes’. While the staging criteria are still becoming widely known, it may be appropriate to refer to these stages as ‘presymptomatic type 1 diabetes’ for some audiences, to highlight that these early stages exist prior to traditional, symptomatic (i.e. stage 3 type 1 diabetes) disease. Individuals with a genetic risk (based on genetic screening and/or family history) or with only single IAb^+^ status have pre-stage 1 type 1 diabetes and can be referred to as ‘at risk’, but individuals with multiple IAb^+^ status are confirmed as having early-stage type 1 diabetes. It must also be clear what the focus of prevention is; for example, prevention of seroconversion, progression to dysglycaemia or of stage 3 type 1 diabetes.

## 2. Partnership between primary-care and specialist HCPs

There is a need for primary care to take on some of the early-stage monitoring and managing IAb^+^ children and adults. However, staging criteria are relatively new and are unlikely to be widely known among primary-care HCPs. Therefore, educational steps and materials must facilitate the partnership between primary-care HCPs and secondary care. Primary-care HCPs in some regions (e.g. the USA, Europe) are involved in screening and monitoring tasks for hypercholesterolaemia and other metabolic syndromes, so the expectation is that this is possible for early-stage type 1 diabetes. A critical need is that all HCPs recognise that some IAb^+^ individuals can progress rapidly, whereas others may not develop symptoms for decades. In this context, the following expert clinical advice is suggested:


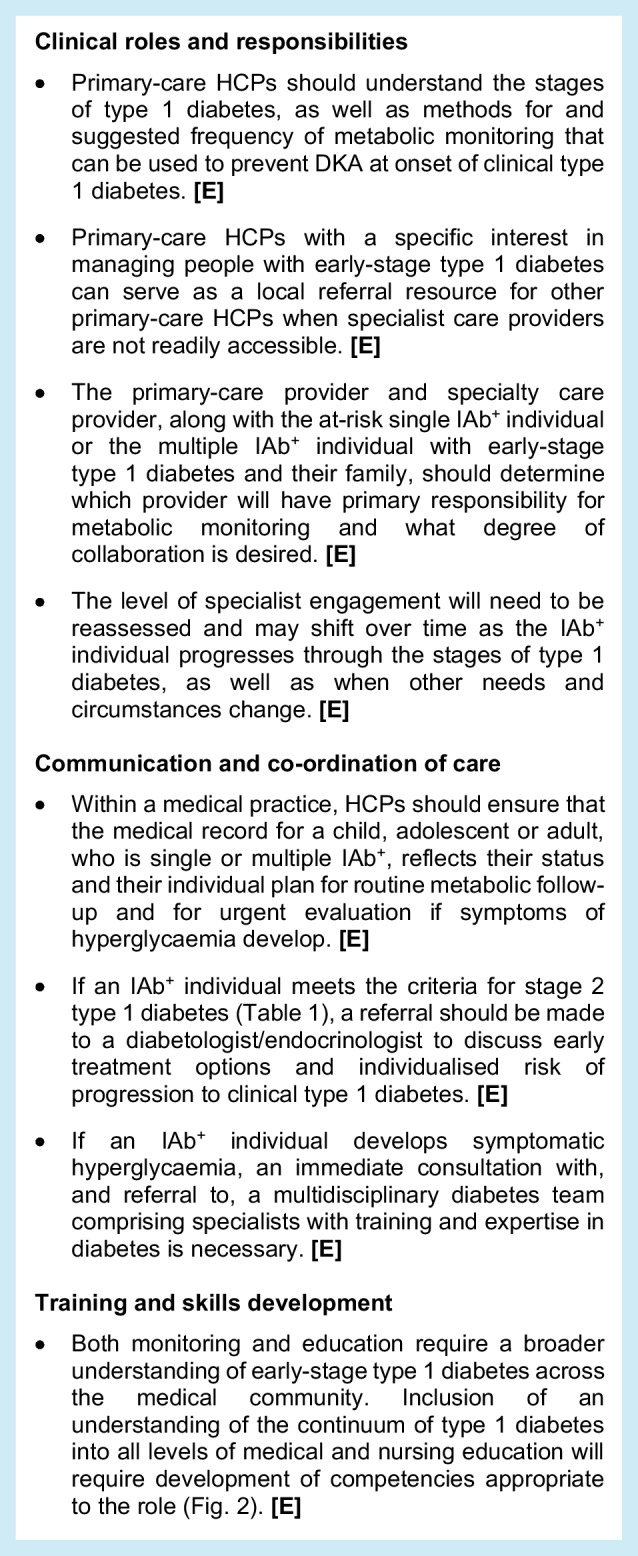


## 3. Monitoring in children and adolescents

### The current landscape of monitoring children and adolescents in early-stage type 1 diabetes

The following section encompasses monitoring of children and adolescents aged up to 17 years. The overall algorithm is summarised in Fig. 1. For a young person who has screened positive for multiple IAb^+^ status, monitoring recommendations are also provided by the ISPAD [16] and the Fr1da study [20].

This expert clinical advice emphasises the need to benchmark the glycaemic stage of disease and to offer ongoing monitoring for disease progression, which should be appropriate to the needs of the affected person and their family. At present, standard 2 h OGTT (1.75 g of glucose per kg of body weight up to 75 g maximum) is the preferred modality, particularly for inclusion in research studies, whereas less-intensive methods are suggested for children or adolescents who decline to undertake OGTT or participate in a research protocol. Even in a clinical-study setting, adherence with OGTT monitoring can be low [76]. Given the diverse settings and resources available, amongst the monitoring tools identified (Table 5), HbA_1c_ testing is not suitable outside of the clinical setting and only random glucose assessments, routine SMBG and CGM, that do not require venipuncture, can be self-managed at home. Studies using CGM in small cohorts of children and youth with stage 1 or stage 2 type 1 diabetes have suggested that glucose levels ≥7.8 mmol/l (≥140 mg/dl) for >10% of each day is associated with an 80% risk of progression to type 1 diabetes within 12 months of the CGM assessment period [72, 77]. In this context, risk of progression to stage 3 type 1 diabetes within 2 years of baseline CGM assessment was 40% in individuals with early-stage type 1 diabetes who spent ≥5% of each day with glucose ≥7.8 mmol/l (≥140 mg/dl) [71]. These outcomes indicate a need for more evidence to confirm the emerging value of CGM in monitoring individuals with early-stage type 1 diabetes and to understand the disease-predictive value of additional CGM metrics. This need is more pressing given that home use of CGM systems and CGM-derived glycaemic metrics is being evaluated for risk stratification for healthy relatives of people with type 1 diabetes [78, 79].

Monitoring at a 6–12 monthly cadence has been used for participants in prevention trials, depending on risk stratification. More-frequent monitoring can be indicated for children who screen positive for islet autoantibodies before 3 years of age and are at high risk of progression [24, 51], for example, at 3- to 6-monthly intervals, depending on staging [24]. It should be noted that, amongst monitoring tools, not all CGM systems are generally available in all regions, or for use in very young children. For all individuals outside of the research setting, reducing the frequency of monitoring can be considered as part of a minimally burdensome approach and modelling studies suggest this can be achieved while meeting the goal of DKA prevention on a population level [80]. In this context, youths of Black race and/or Hispanic ethnicity are less likely to participate with monitoring [81].

### Monitoring for single IAb^+^ at-risk children

Evidence from cohort studies indicates that up to 50% of children with single IAb^+^ status revert to being islet autoantibody negative (IAb^−^) [82, 83]. Children with confirmed persistent single IAb^+^ status are not at high risk for progression compared with those with multiple IAb^+^ status, with one population-based study indicating that the 10 year risk of progression to type 1 diabetes for persistent single IAb^+^ children is 14.5%, with most of that progression (10%) happening in the first 2 years after becoming IAb^+^ [51]. This analysis also showed that the progression rate is higher for young children who have single IA-2A positivity (40.5%), compared with GADA positivity (12.9%) or IAA positivity (13.1%) [51]; however, it must be noted that fewer than 10% of children with single IAb^+^ status are IA-2A^+^. Younger age (<5 years) at first single-confirmed islet autoantibody positivity is a risk factor for progression to multiple islet autoantibody positivity, particularly during the first 2 years after seroconversion [84, 85]. As children age, relative risks for progression with each antibody subtype changes [56], with an increased effect for GADA with increasing age and a reduced effect for IAA [86].

For young children, evidence indicates that metabolic and autoantibody monitoring frequency in the first 2 years after first detection of an autoantibody is key, as this is when spread from at-risk single islet autoantibody positivity to early-stage type 1 diabetes with multiple islet autoantibody positivity is most likely. Following confirmed single IAb^+^ status, the IAb^+^ evolution after 2 years predicts development of clinical type 1 diabetes [87]. Progression to multiple IAb^+^ status or reversion is also highest in the first 2 years in single IAb^+^ pre-school children, with a hazard rate of 0.3 in the first 2 years vs 0.05 for children who have been single IAb^+^ for >2 years [84]. Among children with increased genetic risk, those who remain single IAb^+^ have a risk for type 1 diabetes of 1.8 per 100 person-years, children who revert to negative status have a risk of 0.14 per 100 person-years and children who have never been IAb^+^ have a risk of 0.06 per 100 person-years [83]. The rate of progression to multiple IAb^+^ status also declines with age [88].


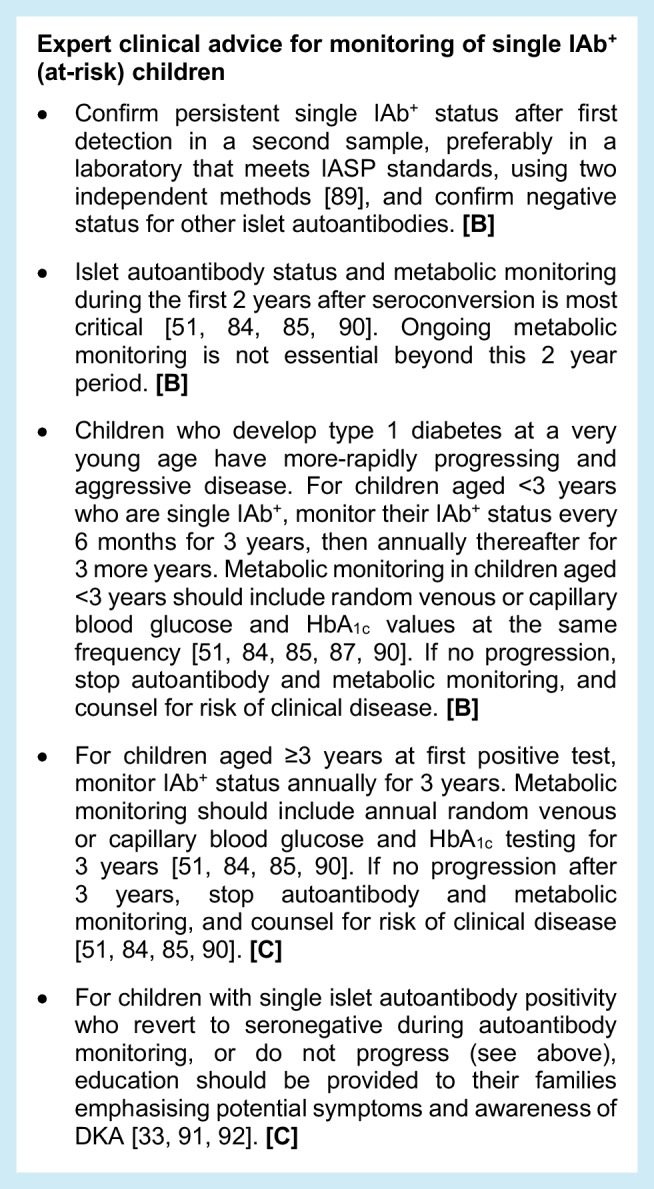


#### Limitation

Many data on single IAb^+^ children are derived from groups with extended prospective follow-up and known genetic risk profiles or first-degree relatives with type 1 diabetes with limited racial/ethnic diversity. Data on individuals in the general population are more limited, particularly in those with a single screening event.

### Monitoring for multiple autoantibody-positive children (early-stage type 1 diabetes)

Children with confirmed multiple IAb^+^ status are at very high risk for progression to stage 3 type 1 diabetes within 15 years. Combined data from five prospective studies indicate that the 15 year risk for stage 3 type 1 diabetes is 85% for children with two islet autoantibodies and 92% for those with three islet autoantibodies, and that there is a >99% lifetime risk [87]. In children with multiple islet autoantibody positivity, younger age at first islet autoantibody detection predicts more-rapid progression to stage 3 type 1 diabetes [51, 93]. Although data on children with multiple islet autoantibody positivity identified from general population screening are derived from shorter follow-up durations, progression rates appear to be similar to those observed in relatives of individuals with type 1 diabetes enrolled in longitudinal research cohort studies [24, 94].

The detection of multiple autoantibodies should be confirmed in a venous sample, within 3 months [49]. However, this should not be a rate-limiting step in the monitoring or treatment process, as progression can happen rapidly in young children. Confirmation is critical, since without it there is a risk of delivering a false diagnosis of multiple IAb^+^ status, with consequent anxiety and distress for the individual. Conversely, although loss of confirmed multiple IAb^+^ status is rare and may be associated with reduced risk of progression to type 1 diabetes [95], monitoring should not be discontinued in this group.

#### Expert clinical advice for monitoring of multiple IAb^+^ children (early-stage type 1 diabetes)

Monitoring of glucose metabolism among children with multiple IAb^+^ status is necessary to predict time to stage 3 diagnosis, identify those who may be eligible for intervention and prevent DKA. Options for metabolic assessments include home SMBG monitoring, periodic CGM assessment, and laboratory testing for HbA_1c_, random venous or capillary blood glucose and OGTT (with stimulated C-peptide assessments). It is acknowledged that there is variable access to high-quality laboratory-testing facilities outside of the research setting. Where possible, the opportunity to undertake monitoring at home or in the primary-care setting should be considered (Table 5).


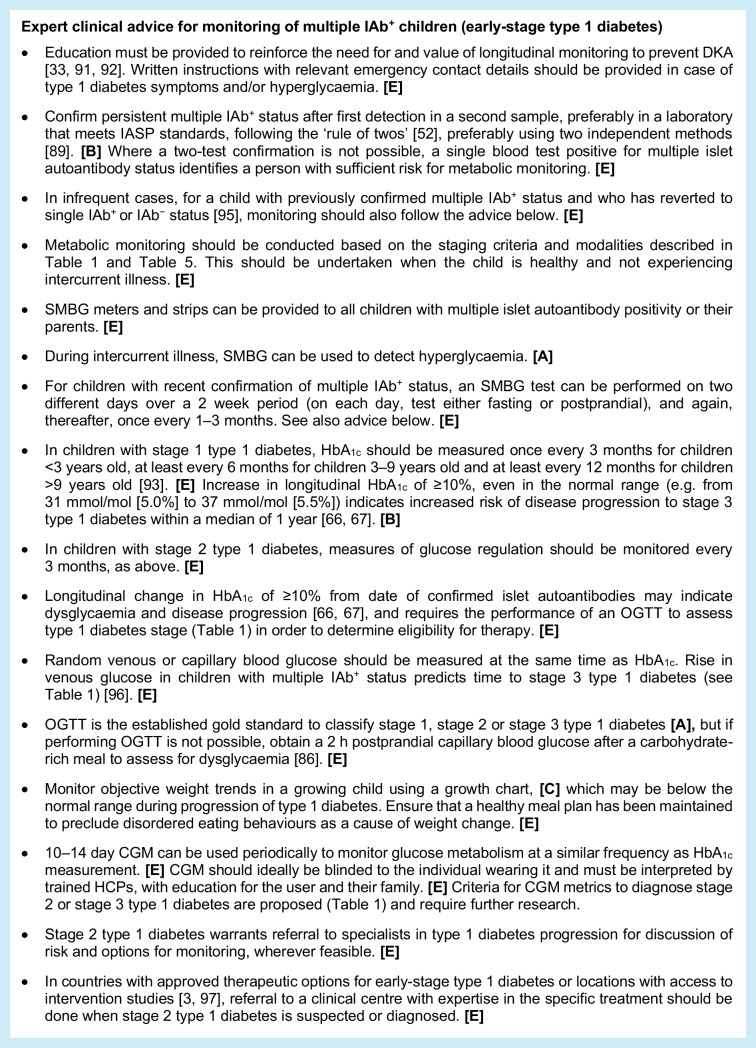


## 4. Monitoring in adults

### The current landscape of monitoring adults who are at risk of or have early-stage type 1 diabetes

The following guidance encompasses monitoring of adults aged 18 years and over, although the advice is based on outcome data that typically reflect adults younger than 45 years of age. Data specific to adults older than this are an important unmet need. Epidemiological data show that, overall, type 1 diabetes is diagnosed more frequently in adulthood than in childhood [98–101], at a median of more than 35 years of age [102, 103]. Despite this, misdiagnoses of type 1 diabetes in adults remain common and are increasingly likely with age [60], setting the scene for development of DKA. In common with childhood-onset type 1 diabetes, adult-onset type 1 diabetes is associated with the presence of islet-specific autoantibodies [104–107]. Although TrialNet cohort data indicate that the rate of progression to type 1 diabetes in IAb^+^ adults is slower than in children, many adults with multiple IAb^+^ status and early-stage type 1 diabetes still develop stage 3 disease [59]. While it has been suggested that progression in some adults may not occur and that some of those who do progress have only single islet autoantibody positivity, further long-term follow-up data are needed to better characterise the long-term implications of persistent autoimmunity in adults [108]. For example, recent data highlight the frequent presence of islet autoimmunity in cohorts presenting with phenotypic type 2 diabetes [109].

Guidance to inform clinical monitoring practices in adults represents a considerable unmet need. There are many evidence-base gaps, including a lack of information about risk of disease progression in IAb^+^ adults without a family history of type 1 diabetes, particularly in individuals with non-European ancestry. Data on suggested monitoring protocols, including effectiveness in preventing DKA and adherence with monitoring, are substantially based on children and adolescents. The frequency of DKA among adults at diagnosis with type 1 diabetes is unknown but believed to be lower than for children, given that adults may recognise and respond to symptoms of hyperglycaemia, and often have higher C-peptide levels at clinical diagnosis and a slower decline in beta cell function over time [110]. Yet, incorrect assumptions leading to underdiagnosis of type 1 diabetes in adults mean many develop DKA before starting insulin therapy.

DKA incidence at clinical diagnosis can be reduced by participation in active monitoring [24, 26, 27]. Regarding frequency of monitoring, modelling based on TrialNet data suggest that conducting approximately half the number of visits involved in a research setting (typically once every 12 months rather than every 6 months), is likely to be effective in substantially reducing the incidence of DKA to the levels seen in research studies both for children and adults [80]. However, data from the TrialNet study indicate that adults 18 years and older are less likely than paediatric participants to engage with recommended monitoring using 6–12 monthly OGTT in the early phases after screening positive for autoantibodies [81]. As with youths, adults of Black race and/or Hispanic ethnicity are less likely to participate with monitoring in this context [81].

Most endocrinologists and primary-care HCPs will not be trained in monitoring adults with single IAb^+^ status or early-stage type 1 diabetes. Thus, the educational need will be significant. As with children and adolescents, monitoring in IAb^+^ adults must be realistic and actionable across diverse regions, with different resources. HCPs are significantly burdened such that additional tasks for monitoring in pre-stage 3 type 1 diabetes must be clinically useful.

### Monitoring for single autoantibody-positive at-risk adults

Frequency of monitoring can be based on the stage at which an individual with islet autoantibody positivity is diagnosed. Single IAb^+^ adults with dysglycaemia should be monitored more frequently than those with normoglycaemia. Additional risk stratification may also be possible based on other characteristics, such as age, or modifiable factors, such as abdominal obesity.


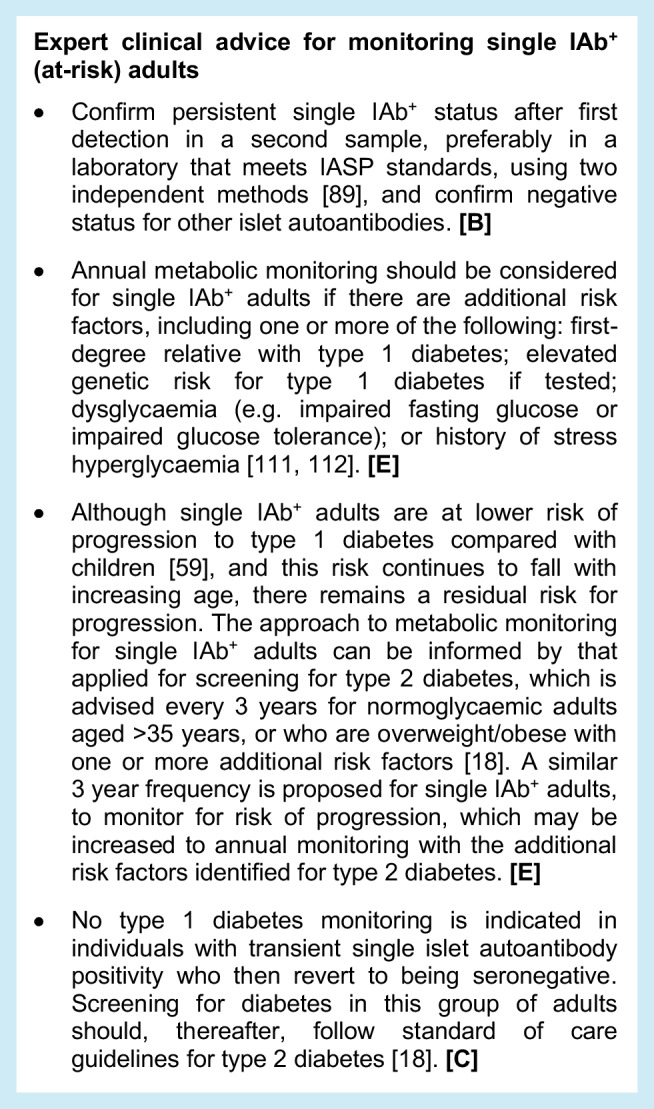


### Monitoring for multiple autoantibody-positive adults (early-stage type 1 diabetes)

As with monitoring in single IAb^+^ adults, more-frequent monitoring is proposed for individuals with multiple IAb^+^ status if they are diagnosed with stage 2 type 1 diabetes compared with stage 1 type 1 diabetes. Risk stratification based on age, abdominal obesity and other modifiable factors also applies.


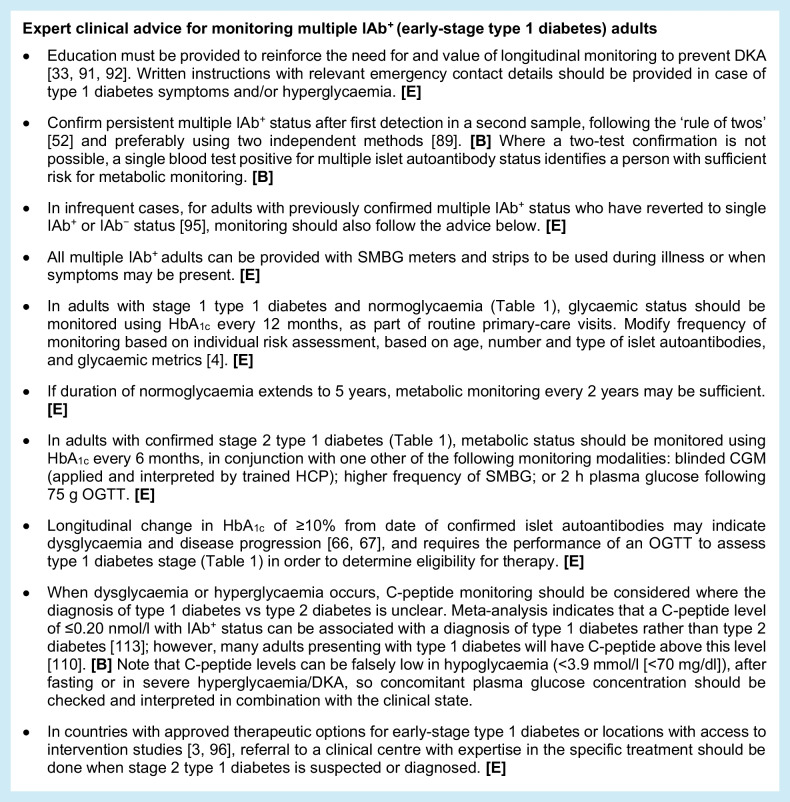


### Monitoring during pregnancy for IAb^+^ women

Evidence on the progression of type 1 diabetes in IAb^+^ pregnant women is limited and research data on this aspect of managing risk in early-stage type 1 diabetes is a significant unmet need (Table 6). With that said, a high risk for postpartum type 1 diabetes has been indicated [114], and the guidance below is primarily based on expert opinion. Pregnancy demands increased pancreatic beta cell function and may result in diabetes, as it does in gestational diabetes mellitus (GDM) [115]. Given that 60% of babies born to women with diagnosed type 1 diabetes are large for gestational age (LGA), which is associated with increased rates of obstetric and neonatal complications [116, 117], it is important to avoid a missed early diagnosis and promote normal fetal development.


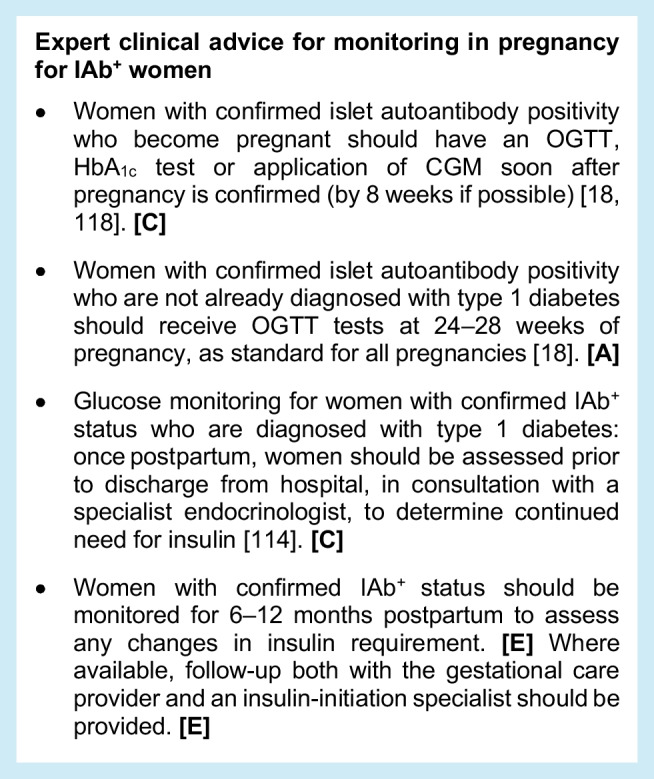


**Table 6:** Selected unmet needs for further research and clinical development

Unmet research needs
• Long-term rates of progression to stage 3 diabetes in IAb^+^ individuals without a family history of T1D, and progression rates in adults and people of non-European ancestry.
• The impact of pregnancy in women who are IAb^+^ and the glycaemic changes that may be evident during pregnancy and in the postpartum period, along with risks for progression to stage 3 T1D during and after pregnancy.
• Neonatal outcomes for infants of women who are IAb^+^ and the association with glycaemic changes during pregnancy.
• Cost-effectiveness of monitoring strategies for individuals with early-stage T1D.
• Timing of insulin initiation in people with presymptomatic T1D, including short- and long-term metabolic and mental-health outcomes of different strategies.
• Impact of education alone, independent of other monitoring activities, on frequency of DKA at diagnosis and presentation of T1D.
• Methods of identifying and monitoring behavioural health needs in early-stage T1D.
Unmet clinical needs
• Comprehensive and consistent educational materials that use consistent language and vocabulary when referring to diabetes stages and risk, including translation into region-specific languages. This applies to all impacted people, from affected individuals to expert providers.
• Validated tools to measure the anxiety, depression and other mental-health behaviours that are specific to early-stage T1D.
• Sufficient availability of mental-health professionals with expertise in T1D, including early-stage T1D in youth and adults.
• Knowledge and coverage of appropriate monitoring by stakeholders (insurers, clinicians, etc.)
• Timely access to expert HCPs and centres of expertise for intervention(s) to delay onset of stage 3 T1D.

The key principles presented in this table and in this consensus document will be subject to updating once additional evidence becomes available

T1D, type 1 diabetes

## 5. When to start insulin

At some point, monitoring will reveal a person with persistent and/or recurrent hyperglycaemia prompting a decision on whether to start insulin, along with associated education and support for affected individuals and their families. As screening programmes identify more people with early-stage type 1 diabetes, more people are being assessed as meeting classic diagnostic criteria for stage 3 type 1 diabetes (Table 1), but who might not yet require insulin therapy. Decisions about how and when to initiate insulin will be based on a range of factors, many of which do not have a body of evidence. Therefore, consideration of starting insulin should trigger a referral to a specialist centre with expertise in initiating and managing people with type 1 diabetes on insulin.

## 6. Education

The primary goals of education for the care of IAb^+^ individuals and their families are outlined in Table 7. Given the paucity of evidence on education for people with early-stage type 1 diabetes, extensive experience in education for stage 3 type 1 diabetes can be extrapolated to this population. National standards for diabetes self-management education and support (DSMES) have been published by the ADA and the Association of Diabetes Care & Education Specialists (ADCES) and are broadly applicable in this context [119]. When appropriate, evidence from studies in stage 3 type 1 diabetes are used to support grading of evidence.

**Table 7:** The primary goals of education for care of IAb^+^ individuals and their families

1. To prevent DKA and promote safe monitoring practices and reduce the occurrence of symptoms of diabetes
2. To minimise the requirement for emergency care, hospital admission and need for intensive care at diagnosis of T1D
3. To improve appropriate risk perception at each monitoring milestone
4. To understand specific outcomes, e.g. prevention of DKA, initiation of insulin therapy
5. To understand available interventions
6. To explore and understand the benefits of individual participation in research studies
7. To provide education that supports psychosocial interventions to optimise general health and mental health for affected individuals and their families

T1D, type 1 diabetes

Experience in clinical studies can also inform education for people with early-stage type 1 diabetes and their families/caregivers. The Environmental Determinants of Diabetes in the Young (TEDDY) prospective study protocol emphasises parental education regarding symptoms and signs of diabetes. For families new to type 1 diabetes, this education provides foundational skills for diabetes management that are a component of reduced parenting stress at the time of stage 3 diagnosis compared with individuals who were members of the community control group and did not receive education [120]. Similarly, families of children with early-stage type 1 diabetes in the Fr1da study are invited to participate in an educational programme of blood glucose monitoring and symptoms of hyperglycaemia/DKA. They are also provided with a guidebook specifically designed for children with early-stage type 1 diabetes, and assigned a contact person to answer questions at any time. Children who take part in this programme alongside metabolic monitoring have a lower rate of DKA and reduced HbA_1c_ at stage 3 type 1 diabetes presentation compared with children who declined education and follow-up [33]. Over 50% had no symptoms at the clinical presentation of stage 3 type 1 diabetes, 93.5% had no weight loss and length of stay in hospital was shorter [91, 92].

Basic community awareness campaigns not associated with monitoring and centred on the early symptoms of type 1 diabetes, that target teachers, paediatricians and parents, have been effective in reducing DKA rates in children in regional settings (Parma in Italy [121] and Newcastle in NSW, Australia [122]). However, national campaigns in Italy and Austria, with the same objectives, have not seen the same impact [123, 124]. The content and delivery of these campaigns were not similar, so it is hard to draw conclusions about the effectiveness of this education.

Education topics and intensity for people with early-stage type 1 diabetes and their families should be based on type 1 diabetes stage, age, rate of progression, etc. First-degree relatives may have different needs for support and guidance from the general population, as they have an established awareness of the implications and impact of IAb^+^ status. Education topics should be linked to specifically timed action plans and include the topics detailed below. Education can be tailored so it is uniquely appropriate for both stage 1 and stage 2 type 1 diabetes (Table 1). Clinical practitioners with experience in early-stage type 1 diabetes should be involved in the later steps of education.

### When should education be provided?

The needs for education are centred on the key moments in the life of the person with early-stage type 1 diabetes [119]. These are at the point of a positive autoantibody screen, at diagnosis of each stage, when monitoring tasks are performed, and annually for review and maintenance. Education is also critical during life transitions and milestones, and when care needs change.

### Key education topics

Education and self-care behaviours for individuals at risk for or with early-stage diabetes (Table 1) can be derived from the overall framework of self-management skills for diabetes and related conditions. These are described in the ADCES7 self-care behaviours [125]. Those relevant for at-risk individuals or those with early-stage type 1 diabetes focus on understanding the implications of their single (at-risk) or multiple (early-stage type 1 diabetes) IAb^+^ status, and the benefits of regular monitoring. Symptom awareness and metabolic monitoring are important to reduce the risks of hospitalisation for DKA. If other family members have type 1 diabetes, HCPs should not assume pre-existing awareness and knowledge. The most-current education should always be mandated.

### Educational topics of highest value for IAb^+^ individuals and family members

For an individual who has tested positive for one or more autoantibodies, a person-centred plan should be developed that is best suited to the IAb^+^ person and their individual situation. Their family members should be included as part of the programme of education. The topics that may have high value are likely to include the following: (1) understanding autoimmunity and the confirmation of single (at-risk) or multiple (early-stage type 1 diabetes) IAb^+^ status; (2) definition of at-risk or early-stage type 1 diabetes; (3) risk perception (accurate risk perception is linked with staying engaged in monitoring and with DKA prevention [126]); (4) risks and benefits of individual participation in research studies; (5) awareness of hyperglycaemia episodes for introducing insulin at the right time; (6) strategies for healthy coping; (7) symptom awareness and prevention of DKA; (8) glucose monitoring (SMBG, CGM), if clinically recommended; (9) healthy behaviours, including meal planning and physical activity; (10) risks and benefits of intervention therapies; (11) monitoring planning, with descriptions of laboratory tests and devices that may be used (Table 5); and (12) treatment options and introduction to insulin therapy.

### Where should education be provided?

Education should be widely accessible via a variety of modalities, across multiple media platforms and settings, and should be crafted with the specific audience’s learning needs in mind. For education aimed at HCPs, a key requirement is for professional associations in all regions to be aligned with the educational programme and curriculum, preferably compatible with their educational platforms and with accreditation. For education aimed at people with pre-stage 3 type 1 diabetes, in-person options associated with clinical appointments or in-group sessions are important, and strong evidence supports DSMES delivery through virtual, telehealth, telephone, text messaging and web-based/mobile phone applications (apps) [127–131].

### Who should provide education?

**Fig. 2:**
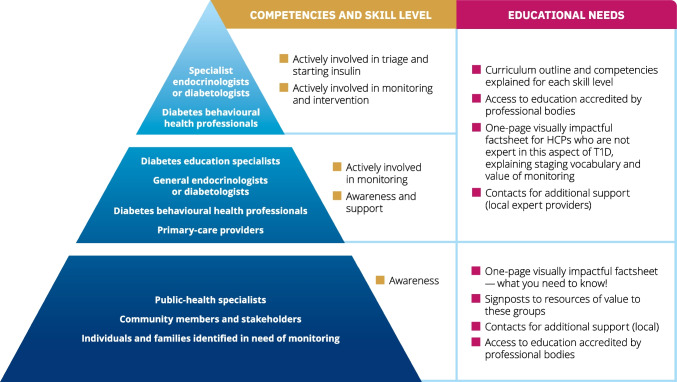
The continuum of educational needs and competencies: what does one need to know? The image represents the anticipated skills that must be developed within the continuum of stakeholders in monitoring presymptomatic type 1 diabetes. The groups indicated within the pyramid sections should have the competencies described and participate as appropriate. The need is for unified, consistent, globally applicable language at all levels. T1D, type 1 diabetes. This figure is available as part of a downloadable slideset

The competencies that must be addressed in education are outlined in Fig. 2. There is a need for diabetes professional associations to endorse the educational goals, educational tools and educational content, as described. Different groups of individuals, including HCPs, community members, and individuals in need of monitoring and their families (indicated in the pyramid sections in Fig. 2), should have the competencies described and participate as appropriate.


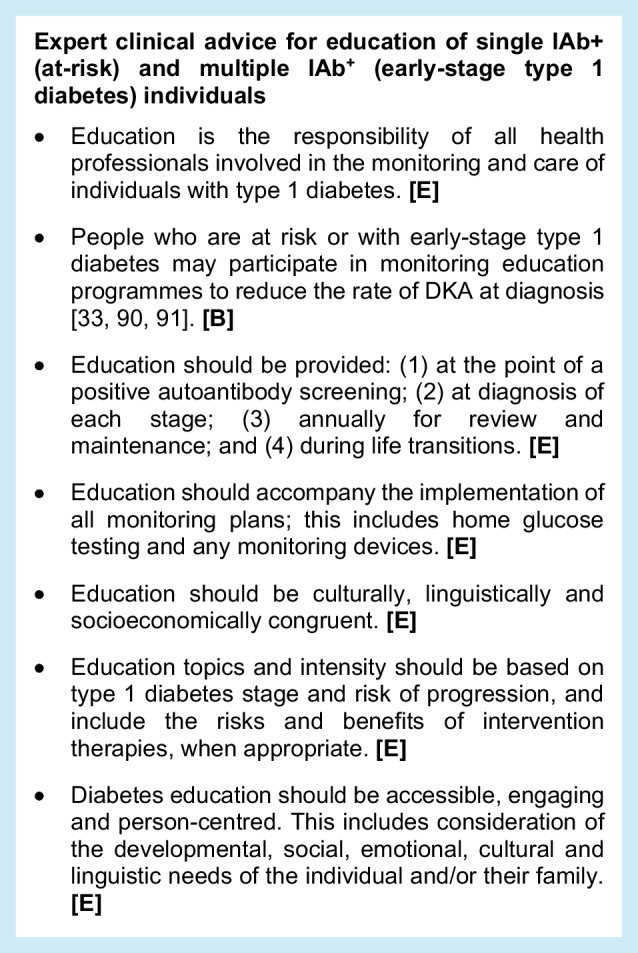


## 7. Psychosocial support

### What is the current landscape regarding psychosocial support for people with type 1 diabetes-related autoantibodies?

People who learn that they or a loved one have type 1 diabetes-related autoantibodies often experience significant stress [132]. This is in part because events that are unpredictable, uncontrollable and threatening may be highly stressful. People who have islet autoantibody positivity, particularly those who have multiple islet autoantibody positivity, will very likely develop type 1 diabetes in the future. However, disease progression is impossible to predict precisely and having IAb^+^ status does not mean imminent type 1 diabetes onset [133, 134]; stage 3 type 1 diabetes, with associated insulin administration and glycaemic monitoring, could be months or even years away [17].

When learning they have type 1 diabetes-related autoantibodies, individuals of all ages and their family members can experience a range of emotional and behavioural reactions [135, 136], including shock, grief, guilt, anger, depression and anxiety. If time passes with no diagnosis of stage 3 type 1 diabetes, cognitions about type 1 diabetes may change and individuals may become convinced they will never get the disease or have reduced risk, despite evidence to the contrary [137]. Parents often engage in behaviours in attempts to prevent type 1 diabetes when faced with the news that their child is at increased risk, even when not provided with recommendations to do so, though more-recent data have shown that lower physical activity and meal plans with a higher glycaemic index are associated with faster progression to type 1 diabetes [138–140]. Meal-planning changes are most commonly reported, with extra monitoring at home (including blood glucose checking) being particularly common in families with someone who already has type 1 diabetes [141, 142].

Research has documented the psychosocial impact of newborn screening [143], as well as genetic and islet autoantibody screening for type 1 diabetes [132, 136]. Failure to understand the screening and risk information presented is common. For example, more than a third of participating mothers and over half of participating fathers in the TEDDY study stated that their child was not at increased risk for type 1 diabetes, despite being clearly informed of their child’s increased genetic risk [137]. To date, no data are available on how children screened positive for islet autoantibodies perceive or react to their risk.

Emotional distress in response to a positive islet autoantibody screen is also common. Many parents of children in the TEDDY study experienced anxiety after learning that their child was at increased risk for developing type 1 diabetes, with mothers reporting higher anxiety than fathers [132]. Although anxiety decreased across time for parents of IAb^+^ children who never developed additional autoantibodies, anxiety remained elevated in many parents of children with multiple autoantibodies for years after the child’s first IAb^+^ test result. Mothers who experienced negative interpersonal life events and postpartum depression, but who were accurate about their child’s type 1 diabetes risk, were particularly vulnerable to heighted anxiety [144]. In the Autoimmunity Screening for Kids (ASK) study, which conducted islet autoantibody screening in the general population, 74.4% of parents reported significant levels of anxiety about their child’s type 1 diabetes risk at the first follow-up visit; parents with lower educational attainment were more likely to exhibit higher levels of anxiety [145].

Around 40% of mothers and 20% of fathers in the Fr1da study reported clinically elevated symptoms of depression after learning that their child was at increased risk for type 1 diabetes as compared with around 18% of mothers and fathers of children who were IAb^−^ [24]. Depressive symptoms declined across 1 year, with scores in mothers of IAb^+^ children remaining slightly elevated as compared with mothers of IAb^−^ children; scores in fathers did not remain elevated.

Although both the ADA and ISPAD have published recommendations about the psychosocial care of individuals with stage 3 type 1 diabetes [146–148], these are limited to general principles for care of those with early-stage type 1 diabetes [149]. Thus, there is an urgent need to provide guidance on psychosocial support for individuals with type 1 diabetes-related autoantibodies and their families.

We recognise regional differences in healthcare resources may limit mental-health resources for care of people with diabetes. In most areas, there are insufficient mental and behavioural health professionals with expertise in the psychosocial aspects of type 1 diabetes who can provide the care recommended by the ADA and ISPAD [146–148].

### What is the purpose of psychosocial support?

The overall goal of providing psychosocial support for individuals identified as having early-stage type 1 diabetes and their families is to assist them in successfully managing the psychosocial impacts associated with this life-changing news. To accomplish this goal, emotional, cognitive and behavioural functioning need to be assessed and addressed, not only in individuals with type 1 diabetes-related autoantibodies but in their family members as well, when appropriate.

### What type of support should be provided?

The essential first step is to ask the individual who is at risk for type 1 diabetes and/or their caregivers and family members about their reactions upon receiving the news that they have type 1 diabetes-related autoantibodies. However, asking once is not enough as adjustment to autoantibody status may change over time [132]. Enquiring about how individuals are coping with the news and their current needs should be conducted at every monitoring visit. Examples of questions to include in the conversation include:

1. How do you feel about this news?
2. Others have said this news brings feelings of sadness or worry, what are your feelings?
3. What is your understanding about having multiple autoantibodies?
4. What type of things are you doing to try to prevent type 1 diabetes?
5. What are your thoughts about talking with a counsellor about your feelings from this news?

Providers can also assess global symptoms of anxiety and depression using age-appropriate standardised and validated questionnaires, such as the Patient Health Questionnaire-9 (PHQ-9) for depression [150] or the Hamilton Anxiety Scale [151]. However, global measures of anxiety and depression may not be sensitive to the emotional impact specifically associated with learning that one—or a loved one—has type 1 diabetes autoantibodies. In such cases, measures that assess emotional reactions to the IAb^+^ status, such as the ‘State’ component of the State-Trait Anxiety Inventory [152] may be more appropriate. At a minimum, HCPs should have conversations with individuals about their reactions to IAb^+^ results rather than relying solely on global measures of psychosocial functioning. Assessments should occur at regular intervals, since reactions are likely to change over time. Additional measures for both depression and anxiety in diabetes are provided in the ADA Psychosocial Care for People With Diabetes Position Statement [147], along with a directory of mental-health providers (https://my.diabetes.org/health-directory [153]).

It is also important to consider developmental and family-specific factors when assessing psychosocial needs. For example, children and adolescents with type 1 diabetes autoantibodies may experience varied emotional, cognitive and behavioural impacts as they develop. This further emphasises the need for ongoing, regular assessment of psychosocial needs. Additionally, individuals with a family history of type 1 diabetes may react differently to learning about type 1 diabetes-related autoantibodies [141] compared with those who are unfamiliar with the disease; family context and prior experience with type 1 diabetes are important considerations when assessing psychosocial impact and the need for additional support.

Although increased anxiety and depression can occur in individuals with type 1 diabetes-related autoantibodies and their family members, this can be reduced by monitoring for the potential development of type 1 diabetes [120]. Providing individuals with regular monitoring for type 1 diabetes, depending on stage, as outlined in earlier sections of this statement, can help individuals manage some of the unpredictability of type 1 diabetes development [120, 132].

Based on the extant literature, diabetes-focused organisations, such as the ADA and ISPAD, have provided recommendations on the importance of individuals with diagnosed type 1 diabetes receiving psychosocial care [146–148] that is preferably integrated into routine diabetes visits and delivered by providers with diabetes-specific training [154]. While the same level of evidence does not yet exist in those individuals with type 1 diabetes-related autoantibodies, the well-documented emotional, cognitive and behavioural impacts of autoantibody status certainly suggest that a similar standard for psychosocial care should be available for all individuals who are at risk for developing type 1 diabetes and their families. For individuals with early-stage type 1 diabetes and their family members, there are well-developed models of managing psychosocial reactions to risk status, including age-specific education and assigned contact people to answer questions, who can also serve as role models [9, 145].

Ideally, psychosocial care should be integrated with routine monitoring visits and delivered by HCPs using a collaborative, person-centred, culturally informed approach. When available, refer to mental and behavioural health professionals with expertise in type 1 diabetes for additional assessment and treatment. For individuals residing in the USA, the ADA publishes the Mental Health Provider Directory, which lists providers with expertise in diabetes [153].


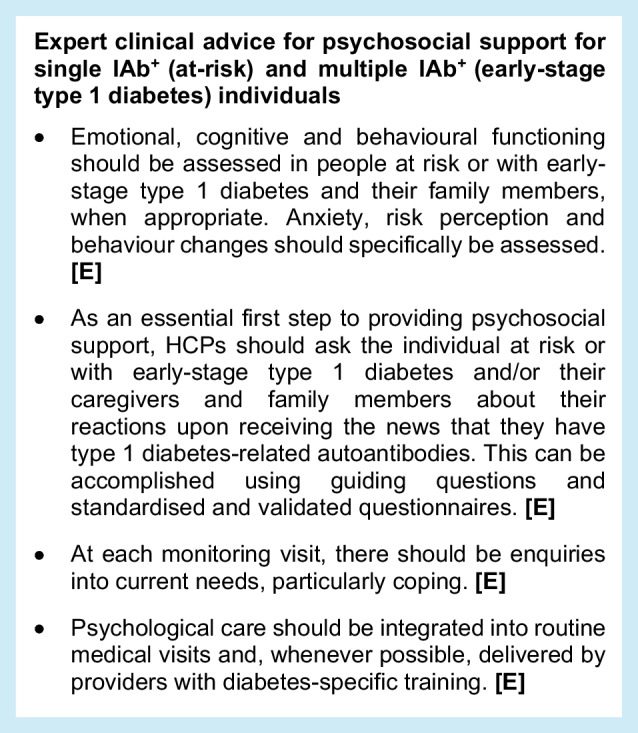


## 8. Unmet needs for further research

This consensus document for monitoring individuals with single (at-risk) and multiple (early-stage type 1 diabetes) islet autoantibody positivity covers key principles based on existing evidence and agreed expert opinion. It also highlights the significant unmet need for further research on early-stage type 1 diabetes to further increase the rigour for future guidance and recommendations, and drive the evolution of clinical care for people who have tested positive for islet autoantibodies. The key principles in this consensus document will be subject to updating once additional evidence becomes available, as indicated in Table 6.

## Supplementary Information

Below is the link to the electronic supplementary material.

ESM Table (PDF 123 KB)

Slideset of figures (PPTX 277 KB)

## Abbreviations

ADCES: Association of Diabetes Care & Education Specialists
CGM: Continuous glucose monitoring
DCES: Diabetes care and education specialists
DKA: Diabetic ketoacidosis
DSMES: Diabetes self-management education and support
HCP: Healthcare professional
IAb^−^: Islet autoantibody negative
IAb^+^: Islet autoantibody positive
IASP: Islet Autoantibody Standardisation Program
ISPAD: International Society for Pediatric and Adolescent Diabetes
SMBG: Self-monitored blood glucose
TEDDY: The Environmental Determinants of Diabetes in the Young
