# Correction to: Consensus guidance for monitoring individuals with islet autoantibody‑positive pre‑stage 3 type 1 diabetes

**DOI:** 10.1007/s00125-024-06266-6

**Published:** 2024-09-04

**Authors:** Moshe Phillip, Peter Achenbach, Ananta Addala, Anastasia Albanese‑O’Neill, Tadej Battelino, Kirstine J. Bell, Rachel E. J. Besser, Ezio Bonifacio, Helen M. Colhoun, Jennifer J. Couper, Maria E. Craig, Thomas Danne, Carine de Beaufort, Klemen Dovc, Kimberly A. Driscoll, Sanjoy Dutta, Osagie Ebekozien, Helena Elding Larsson, Daniel J. Feiten, Brigitte I. Frohnert, Robert A. Gabbay, Mary P. Gallagher, Carla J. Greenbaum, Kurt J. Griffin, William Hagopian, Michael J. Haller, Christel Hendrieckx, Emile Hendriks, Richard I. G. Holt, Lucille Hughes, Heba M. Ismail, Laura M. Jacobsen, Suzanne B. Johnson, Leslie E. Kolb, Olga Kordonouri, Karin Lange, Robert W. Lash, Åke Lernmark, Ingrid Libman, Markus Lundgren, David M. Maahs, M. Loredana Marcovecchio, Chantal Mathieu, Kellee M. Miller, Holly K. O’Donnell, Tal Oron, Shivajirao P. Patil, Rodica Pop‑Busui, Marian J. Rewers, Stephen S. Rich, Desmond A. Schatz, Rifka Schulman‑Rosenbaum, Kimber M. Simmons, Emily K. Sims, Jay S. Skyler, Laura B. Smith, Cate Speake, Andrea K. Steck, Nicholas P. B. Thomas, Ksenia N. Tonyushkina, Riitta Veijola, John M. Wentworth, Diane K. Wherrett, Jamie R. Wood, Anette‑Gabriele Ziegler, Linda A. DiMeglio

**Affiliations:** 1 Institute for Endocrinology and Diabetes, National Center for Childhood Diabetes, Schneider Children’s Medical Center of Israel, Petah Tikva, Israel; 2 Faculty of Medical and Health Sciences, Tel Aviv University, Tel Aviv, Israel; 3 Institute of Diabetes Research, Helmholtz Zentrum Munchen, German Research Center for Environmental Health, Munich‑Neuherberg, Germany; 4 Forschergruppe Diabetes, Technical University Munich, Klinikum Rechts Der Isar, Munich, Germany; 5 Division of Endocrinology, Department of Pediatrics, Stanford University School of Medicine, Stanford, CA USA; 6 Stanford Diabetes Research Center, Stanford University School of Medicine, Stanford, CA USA; 7 Breakthrough T1D, Gainesville, FL USA; 8 Faculty of Medicine, University of Ljubljana, Ljubljana, Slovenia; 9 Department of Endocrinology, Diabetes and Metabolism, University Medical Centre Ljubljana, Ljubljana, Slovenia; 10 Charles Perkins Centre and Faculty of Medicine and Health, University of Sydney, Sydney, NSW Australia; 11 JDRF/Wellcome Diabetes and Inflammation Laboratory, Wellcome Centre Human Genetics, Nuffield Department of Medicine Oxford NIHR Biomedical Research Centre, University of Oxford, Oxford, UK; 12 Department of Paediatrics, University of Oxford, Oxford, UK; 13 Center for Regenerative Therapies Dresden, Faculty of Medicine, Technical University of Dresden, Dresden, Germany; 14 Paul Langerhans Institute Dresden, Helmholtz Centre Munich at the University Clinic Carl Gustav Carus of TU Dresden and Faculty of Medicine, Dresden, Germany; 15 The Institute of Genetics and Cancer, University of Edinburgh, Edinburgh, UK; 16 Department of Public Health, NHS Fife, Kirkcaldy, UK; 17 Robinson Research Institute, The University of Adelaide, Adelaide, SA Australia; 18 Adelaide Medical School, The University of Adelaide, Adelaide, SA Australia; 19 Division of Paediatrics, Women’s and Children’s Hospital, Adelaide, SA Australia; 20 Discipline of Paediatrics & Child Health, School of Clinical Medicine, UNSW Medicine & Health, Sydney, NSW Australia; 21 Breakthrough T1D, Lisbon, Portugal; 22 International Society for Pediatric and Adolescent Diabetes (ISPAD), Berlin, Germany; 23 Diabetes & Endocrine Care Clinique Pediatrique (DECCP), Clinique Pediatrique/Centre Hospitalier (CH) de Luxembourg, Luxembourg City, Luxembourg; 24 Faculty of Science, Technology and Medicine, University of Luxembourg, Esch‑Belval, Luxembourg; 25 Department of Pediatrics, Barbara Davis Center for Diabetes, University of Colorado Anschutz Medical Campus, Aurora, CO USA; 26 Department of Clinical and Health Psychology, University of Florida, Gainesville, FL USA; 27 Department of Pediatrics, University of Florida Diabetes Institute, Gainesville, FL USA; 28 Breakthrough T1D, New York, NY USA; 29 T1D Exchange, Boston, MA USA; 30 Department of Clinical Sciences, Malmo, Lund University, Lund, Sweden; 31 Department of Pediatrics, Skane University Hospital, Malmo and Lund, Sweden; 32 Children’s Diabetes Foundation, Aurora, CO USA; 33 American Diabetes Association, Arlington, VA USA; 34 NYU Langone Medical Center, New York, NY USA; 35 Center for Interventional Immunology and Diabetes Program, Benaroya Research Institute, Seattle, WA USA; 36 Sanford Research, Sioux Falls, SD USA; 37 Department of Pediatrics, Sanford School of Medicine, University of South Dakota, Sioux Falls, SD USA; 38 Pacific Northwest Diabetes Research Institute, University of Washington, Seattle, WA USA; 39 Division of Endocrinology, University of Florida College of Medicine, Gainesville, FL USA; 40 School of Psychology, Deakin University, Geelong, VIC Australia; 41 The Australian Centre for Behavioural Research in Diabetes, Diabetes Victoria, Carlton, VIC Australia; 42 Institute for Health Transformation, Deakin University, Geelong, VIC Australia; 43 Department of Paediatrics, University of Cambridge and Cambridge University Hospitals NHS Foundation Trust, Cambridge Biomedical Campus, Cambridge, UK; 44 Human Development and Health, Faculty of Medicine, University of Southampton, Southampton, UK; 45 National Institute for Health and Care Research Biomedical Research Centre, University Hospital Southampton NHS Foundation Trust, Southampton, UK; 46 Mount Sinai South Nassau, Oceanside, NY USA; 47 Department of Pediatrics, Indiana University School of Medicine, Indianapolis, IN USA; 48 Department of Behavioral Sciences and Social Medicine, Florida State University College of Medicine, Tallahassee, FL USA; 49 Association of Diabetes Care & Education Specialists, Chicago, IL USA; 50 Medical Psychology, Hannover Medical School, Hannover, Germany; 51 Endocrine Society, Washington, DC USA; 52 Division of Pediatric Endocrinology and Diabetes, University of Pittsburgh, UPMC Children’s Hospital of Pittsburgh, Pittsburgh, PA USA; 53 Department of Pediatrics, Kristianstad Hospital, Kristianstad, Sweden; 54 Department of Pediatrics, Cambridge University Hospitals NHS Foundation Trust, Cambridge, UK; 55 Department of Endocrinology, UZ Gasthuisberg, KU Leuven, Leuven, Belgium; 56 Department of Family Medicine, Brody School of Medicine, East Carolina University, Greenville, NC USA; 57 Department of Internal Medicine, Division of Metabolism, Endocrinology and Diabetes, University of Michigan, Ann Arbor, MI USA; 58 Center for Public Health Genomics, University of Virginia, Charlottesville, VA USA; 59 Department of Pediatrics, University of Florida, Gainesville, FL USA; 60 Division of Endocrinology, Long Island Jewish Medical Center, Northwell Health, Donald and Barbara Zucker School of Medicine at Hofstra/Northwell, New Hyde Park, NY USA; 61 Division of Pediatric Endocrinology and Diabetology, Herman B Wells Center for Pediatric Research, Center for Diabetes and Metabolic Diseases, Indiana University School of Medicine, Indianapolis, IN USA; 62 Diabetes Research Institute, University of Miami Miller School of Medicine, Miami, FL USA; 63 Cincinnati Children’s Hospital Medical Center, Cincinnati, OH USA; 64 NIHR Clinical Research Network Thames Valley and South Midlands, Oxford, UK; 65 Division of Endocrinology and Diabetes, Baystate Children’s Hospital and University of Massachusetts Chan Medical School - Baystate, Springfield, MA USA; 66 Research Unit of Clinical Medicine, Department of Pediatrics, Medical Research Center, Oulu University Hospital and University of Oulu, Oulu, Finland; 67 The Walter and Eliza Hall Institute of Medical Research, Parkville, VIC Australia; 68 Department of Diabetes and Endocrinology, Royal Melbourne Hospital, Parkville, VIC Australia; 69 Department of Paediatrics, The Hospital for Sick Children, University of Toronto, Toronto, ON Canada; 70 Department of Pediatric Endocrinology, Rainbow Babies and Children’s Hospital, University Hospitals Cleveland Medical Center, Cleveland, OH USA

**Correction to: Diabetologia**

10.1007/s00125-024-06205-5

Unfortunately, the affiliation for Linda A. DiMeglio was incorrect in this paper. The original article has been corrected.

