# Supplementary material for: Consensus guidance for monitoring individuals with islet autoantibody-positive pre-stage 3 type 1 diabetes
Source: Diabetologia. 2024 Jun 24;67(9):1731–59. doi: 10.1007/s00125-024-06205-5 (PMC11410955; doi:10.1007/s00125-024-06205-5)
Supplement: Supplementary file 1 — ESM Table (PDF 123 KB) [file 125_2024_6205_MOESM1_ESM.pdf]

Supplementary materials

Consensus guidance for monitoring individuals with islet autoantibody-positive pre-stage 3 type 1 diabetes

Moshe Phillip et al

ESM Table 1. ADA evidence-grading system for Standards of Care in Diabetes*

Level of evidence	Description
**A**	Clear evidence from well-conducted, generalizable randomized controlled trials that are adequately powered, including:<br>• Evidence from a well-conducted multicenter trial<br>• Evidence from a meta-analysis that incorporated quality ratings in the analysis<br><br>Supportive evidence from well-conducted randomized controlled trials that are adequately powered, including:<br>• Evidence from a well-conducted trial at one or more institutions<br>• Evidence from a meta-analysis that incorporated quality ratings in the analysis
**B**	Supportive evidence from well-conducted cohort studies<br>• Evidence from a well-conducted prospective cohort study or registry<br>• Evidence from a well-conducted meta-analysis of cohort studies<br><br>Supportive evidence from a well-conducted case-control study
**C**	Supportive evidence from poorly controlled or uncontrolled studies<br>• Evidence from randomized clinical trials with one or more major or three or more minor methodological flaws that could invalidate the results<br>• Evidence from observational studies with high potential for bias (such as case series with comparison with historical controls)<br>• Evidence from case series or case reports<br><br>Conflicting evidence with the weight of evidence supporting the recommendation
**E**	Expert consensus or clinical experience

* Reproduced from [75], American Diabetes Association, 2023. Copyright and all rights reserved. Material from this publication has been used with the permission of American Diabetes Association.
