# Supplementary material for: Consensus guidance for monitoring individuals with islet autoantibody-positive pre-stage 3 type 1 diabetes
Source: Diabetologia. 2024 Jun 24;67(9):1731–59. doi: 10.1007/s00125-024-06205-5 (PMC11410955; doi:10.1007/s00125-024-06205-5)
Supplement: Supplementary file 2 — Slideset of figures (PPTX 277 KB) [file 125_2024_6205_MOESM2_ESM.pptx]

## Slide 1
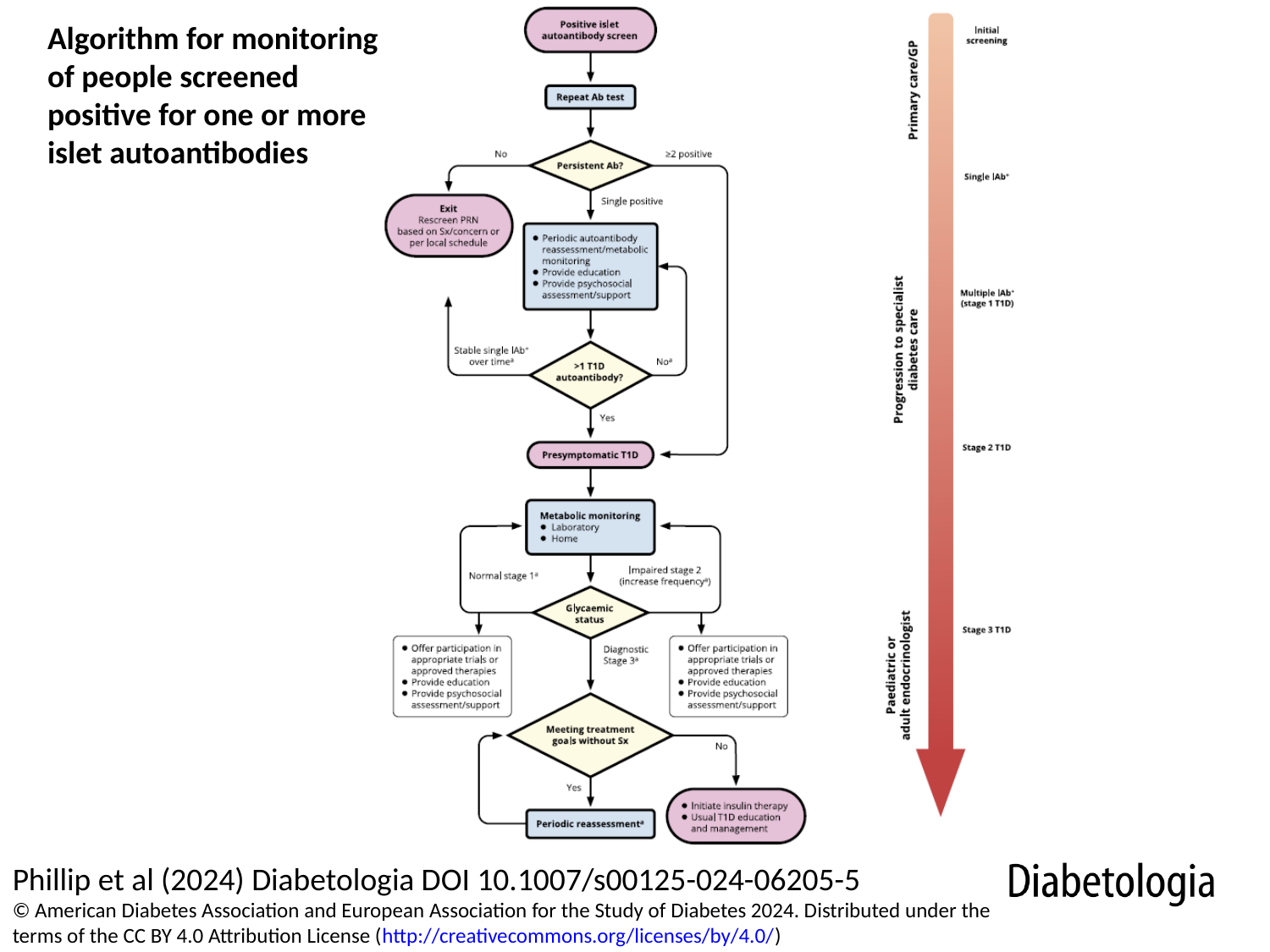

Algorithm for monitoring of people screened positive for one or more islet autoantibodies
Phillip et al (2024) Diabetologia DOI 10.1007/s00125-024-06205-5
© American Diabetes Association and European Association for the Study of Diabetes 2024. Distributed under the terms of the CC BY 4.0 Attribution License (http://creativecommons.org/licenses/by/4.0/)

## Slide 2
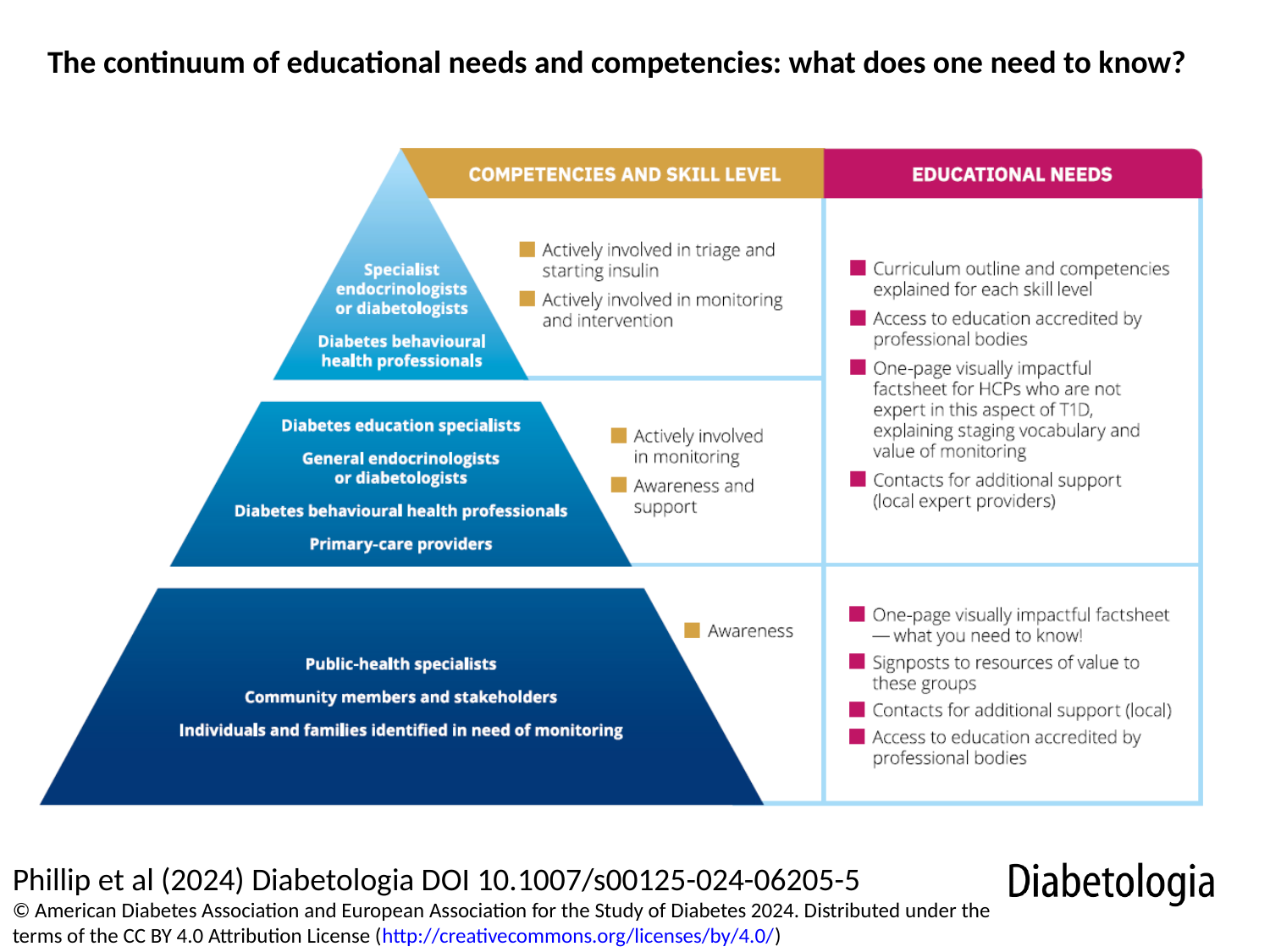

The continuum of educational needs and competencies: what does one need to know?
Phillip et al (2024) Diabetologia DOI 10.1007/s00125-024-06205-5
© American Diabetes Association and European Association for the Study of Diabetes 2024. Distributed under the terms of the CC BY 4.0 Attribution License (http://creativecommons.org/licenses/by/4.0/)
